# Multiplexed DNA detection based on positional encoding/decoding with self-assembled DNA nanostructures†
†Electronic supplementary information (ESI) available: Experimental details, additional DNA detection figures, and DNA sequence information. See DOI: 10.1039/c4sc02696a



**DOI:** 10.1039/c4sc02696a

**Published:** 2014-10-15

**Authors:** Sha Sun, Huaxin Yao, Feifei Zhang, Jin Zhu

**Affiliations:** a Department of Polymer Science and Engineering , School of Chemistry and Chemical Engineering , State Key Laboratory of Coordination Chemistry , Nanjing National Laboratory of Microstructures , Nanjing University , Nanjing 210093 , China . Email: jinz@nju.edu.cn

## Abstract

Current multiplexed analysis methods suffer from either slow reaction kinetics (planar arrays) or complicated encoding/decoding procedures (suspension arrays). We report herein a multiplexed DNA detection strategy that addresses these issues, based on positional encoding/decoding with self-assembled DNA nanostructures. The strategy enables the acquisition of high-resolution, consistent, and quantitative assay results in a single round of a transmission electron microscopy imaging operation. Applications in polymerase chain reaction-free settings and assays of other structurally distinct targets can be anticipated through the implementation of the strategy with miniaturized femtoliter/attoliter dispensing technology and readily accessible DNA conjugate structures.

## Abstract 2

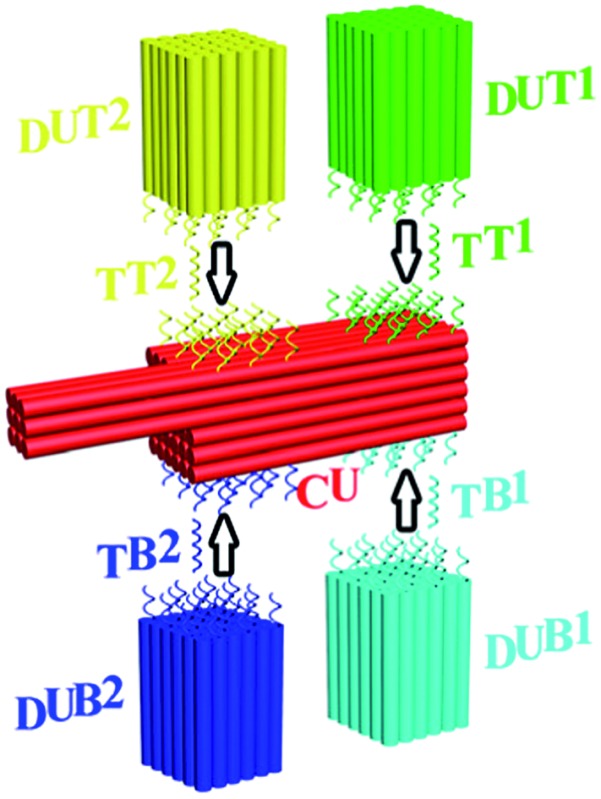
A multiplexed DNA detection strategy with fast hybridization kinetics based on positional encoding/decoding with self-assembled DNA nanostructures has been developed.

## Introduction

**Fig. 1:**
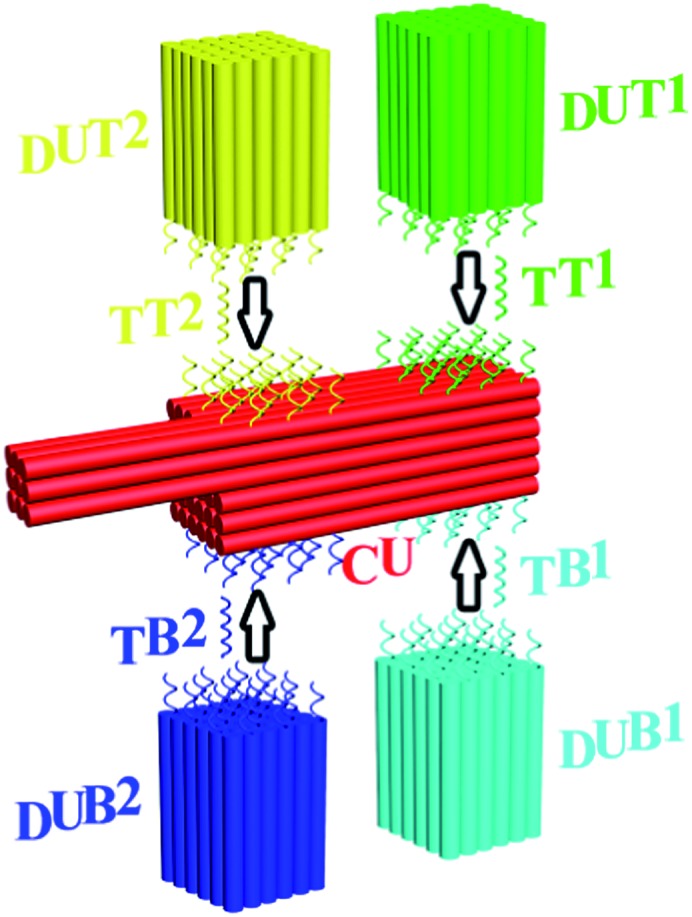
Schematic illustration of the PED-SADNA strategy. A CU is designed and assembled for the positional encoding, featuring a core cuboid with binding sites for multiple targets (TT1, TT2, TB1, TB2) and a registry marker at the corner as a reference for the differentiation of both lateral surfaces (top surface for TT1 and TT2, bottom surface for TB1 and TB2) and longitudinal positions (remote positions for TT1 and TB1, proximal positions for TT2 and TB2) where those sites are located. Accordingly, multiple DU (DUT1, DUT2, DUB1, DUB2) containing sequences that can hybridize with the remaining portions of corresponding targets are individually assembled. Target binding will direct the respective DU to the partner CU site. Staining with uranyl formate and TEM visualization allow positional decoding and unambiguous identification of target DNA.

The ability to detect multiple biologically relevant species in parallel allows the utilization of molecular profiling as an elaborate tool for the elucidation of biological phenomena. Such an information-intensive multiplexed analysis process has been enabled by two distinct assay formats: planar arrays^1^ and suspension arrays.^2^ Planar arrays provide a convenient readout of targets through the positional encoding/decoding strategy, where the location of each spot in an ordered two-dimensional pattern defines the identity of a prospective target. This assay scheme is conceptually straightforward but suffers from slow reaction kinetics.^1d–g^ Suspension arrays exhibit fast reaction kinetics, but both of the encoding and decoding processes are generally rather complicated. An assay system that combines facile positional encoding/decoding capability and fast reaction kinetics would be ideal for efficient extraction of biological information.^3^ Herein we report a multiplexed DNA detection strategy based on positional encoding/decoding with self-assembled DNA nanostructures (PED-SADNA) (Fig. 1). Specifically, a self-assembled three-dimensional core DNA structure^4^ with a registry marker asymmetrically positioned at one end and multiple types of target-binding capture probe sequences placed at regular intervals (collectively termed chip unit, or CU, by analogy to the planar DNA chip) is fabricated for the unambiguous positional encoding of DNA target information. Accordingly, multiple satellite DNA structures containing detection probe sequences (termed detection unit, or DU) for the remaining portions of corresponding targets are individually assembled. The presence of each target will direct the respective DU to the partner site of CU through hybridization. Staining of DNA nanostructures with uranyl formate and visualization with transmission electron microscopy (TEM) allow positional decoding and unambiguous identification of target DNA.

Although self-assembled DNA nanostructures have been previously used for molecular diagnostics, they face major challenges with respect to the signal readout.^5,6^ Atomic force microscopy^5,7^ allows high-resolution imaging down to the single-molecule level under optimized conditions, but the exact surface feature observed depends heavily on technical variables of a particular operation (*e.g.* expertise of operating personnel, morphology of scanning tip). Fluorescence microscopy^6,8^ provides multi-color imaging capability, but limited spatial resolution and requirement for multiple imaging cycles/subsequent superposition processes (for the generation of pseudo-color images through multiple excitation source wavelengths or DNA strand exchange) poses significant restrictions on this sequential multiplexing methodology. The PED-SADNA method reported herein offers a robust solution to the above issues and delivers high-resolution, consistent, and quantitative assay results in a single round of a TEM imaging operation.

## Results and discussion

The asymmetric design of CU enables positional encoding at both lateral and longitudinal directions through the differentiation of surfaces and positions where the capture probes are located. The proof-of-concept CU system reported herein is comprised of a 6H (helices)/6H/128BP (base pairs) (all DNA sequences for self-assembly were designed with program Sequin^9^ with a criton size of 7) square lattice core cuboid, which translates to a length scale of 15 nm/15 nm/43 nm, and a 3H/3H/64BP registry marker at a corner. The longitudinal dimension of the core cuboid and corner arrangement of the registry marker give a positional encoding capacity of four (two encoding positions for each surface × two surfaces). Accordingly, four types of capture probe sequences, each 11 nt (nucleotides) in length and in 14 copies (with the end of core cuboid opposite to the registry marker counted as 0 BP, the locations of two capture probes are: 3 strands at 8 BP, 3 at 16 BP, 2 at 24 BP, 3 at 40 BP and 3 at 48 BP; and the locations of two other capture probes are: 3 at 88 BP, 3 at 104 BP, 2 at 112 BP, 3 at 120 BP and 3 at 128 BP) specifically targeting a 26 nt sequence, were integrated into the design of the CU system. The gap distance between the two encoding sites on one surface is 13.5 nm, which can be readily resolved by TEM. For each of these targets, 15 copies of a 15 nt detection probe sequence were engineered into a 6H/6H/64BP DU framework. Positional readout of the CU–DU hybrid allows the extraction of target sequence information. In the CU/DU detection system reported herein, the location distributions of four target-binding sites in CU as described above secure a similar hybridization efficiency for each target. The size dimension of CU enables the docking of the CU–DU hybrid in a desired side-on orientation on the TEM grid, which allows straightforward visualization of DU.

**Fig. 2:**
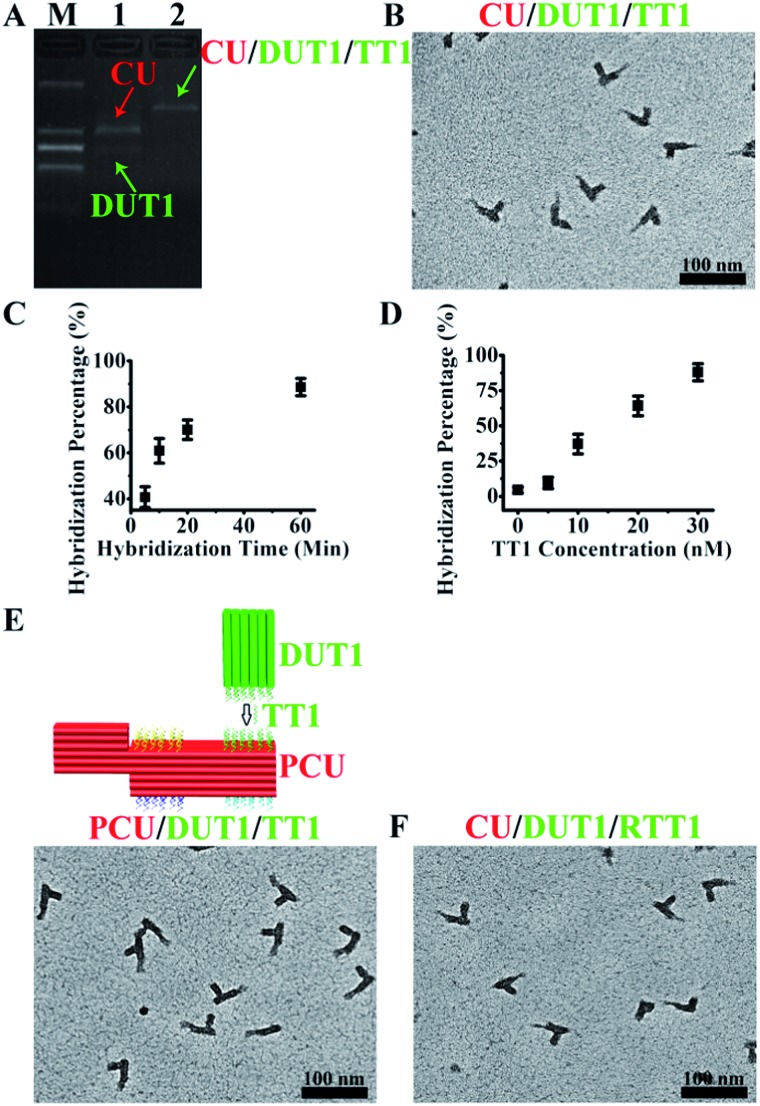
Single-target DNA and RNA detection with PED-SADNA. (A) Gel electrophoresis bands of CU and DUT1 in the absence (lane 1) and presence (lane 2) of target DNA TT1 (lane M represents the molecular weight marker). (B) TEM image of CU and DUT1 in the presence of target DNA TT1. (C) Hybridization percentage of CU and DUT1 in the presence of target DNA TT1 as a function of hybridization time. (D) Hybridization percentage of CU and DUT1 in the presence of target DNA TT1 as a function of concentration. (E) Schematic illustration and TEM image of PCU and DUT1 in the presence of target DNA TT1. (F) TEM image of CU and DUT1 in the presence of target RNA RTT1.

We commenced the evaluation of the feasibility of our detection system with a single-target (TT1) sample. This demands the fabrication and purification of the corresponding CU and DUT1. These tailorable CU and DU can be conveniently synthesized by slow annealing of hundreds of single-strand DNA (ssDNA) sequences and subsequent purification with gel electrophoresis. A commonly observed phenomenon for self-assembled DNA nanostructures is undesired multimerization, especially after their extended storage at 4 °C. As such, before purification, a 4 h pre-heating step at 37 °C for the annealing product is needed for the increase of discrete cuboid recovery yield. Experiments with other alternative purification methods prove to be not as convenient and effective as gel electrophoresis. Electro-elution^10^ allows purification but involves extra solution exchange steps, and direct ultra-filtration^5a,11^ can not completely eliminate excess ssDNA. In TEM imaging, instead of using glow discharge^12^ for the creation of negative staining, we intentionally add a ssDNA sequence, which does not interfere with the assay, as a means of generating a hydrophilic TEM grid surface^13^ for a convenient positive staining of CU and DU. Without assistance from such a ssDNA, limited access to aqueous solution due to the hydrophobicity of the TEM grid results in only partial staining and damaged outlook for CU and DU. This positive staining method provides such a high contrast for DNA nanostructures that we could image them easily in several minutes. With 5 nM each of purified CU and DUT1 placed in the detection system, the presence of TT1 (300 nM) leads to the formation of a lower mobility band in gel electrophoresis (Fig. 2A) and TEM imaging confirmed the generation of CU–DUT1 at the desired location (Fig. 2B). Consistent with the expected existence of a size effect for the docking orientation of CU on the TEM grid, an important experimental observation is that CU with a core cuboid size of 6H/6H/128BP can achieve the desired side-on settlement, whereas a switch of its core cuboid to 6H/6H/64BP renders the majority of CU in head-on settlement, whether being alone or in the form of a CU–DU hybrid. A screening of Mg^2+^ concentration indicates that hybridization proceeds efficiently at 11 mM. Statistical analysis by manual counting of each structurally resolved CU reveals a hybridization percentage (HP) of ∼41% (defined as the percentage of observed CU–DUT1 over all CU) after merely 5 min and ∼89% (a plateau value comparable to that reported previously on DNA tiles^5*a*^) after 1 h (Fig. 2C). Therefore, essentially, the whole assay can be accomplished within 74 min (1 h hybridization, 2 min loading of hybridization product onto the TEM grid, 2 min staining, and 10 min TEM imaging). A longer duration of hybridization (8 h) provides essentially identical HP (∼91%). Titration of TT1 shows a positive correlation of HP with TT1 concentration over the range of 10–30 nM, providing the possibility of target quantification (Fig. 2D), and the HP at 30 nM (∼89%) has reached the plateau value. It should be noted that DUT1 can be occasionally identified at a location other than that targeted, as has also been observed previously in other self-assembled DNA systems.^6*a*^ This “misplacement” of DUT1, due to either accidental proximal settlement of CU and DUT1 on the TEM grid or non-specific interaction, can be counted as the background in our assay system. Indeed, such a phenomenon occurs even in the absence of a target DNA. The unoptimized detection limit therefore currently stands at 10 nM (HP ∼37%, compared with a background value of ∼4%, Fig. 2D). The ultimate measure of the assay sensitivity of a diagnostic method, in terms of absolute target quantity, is also dictated by the sample volume. In this regard, a major advantage of the PED-SADNA strategy reported herein is the ability to scale down the sample volume without affecting the assay quality because of the high resolving power of TEM. When combined with the recently developed femtoliter/attoliter dispensing technology,^14^ we anticipate that our detection system can be routinely applied in polymerase chain reaction (PCR, an amplification method that suffers from sensitivity to contamination and faces major issues in terms of multiplexing^15^)-free settings. Overall, although both DNA self-assembly and TEM imaging seem, at first glance, inconvenient for the implementation of an assay tool, recent advances in both fields^4,16,17^ with respect to automation and versatility render the PED-SADNA strategy demonstrated herein highly practical.

The modular design of self-assembled DNA nanostructures allows the variation of registry marker to a protruded geometry (3H/6H/64BP) as an equally effective indicator of orientation for such a variant configuration, PCU (Fig. 2E). Also, the CU/DU assay system can be adapted to the detection of other types of targets as long as corresponding target-binding probes are in place. RNA exists in a variety of forms and is an important diagnostic and therapeutic target. Indeed, the CU/DUT1 design allows its direct application in the detection of a 26 nt RNA target (RTT1) (Fig. 2F).

**Fig. 3:**
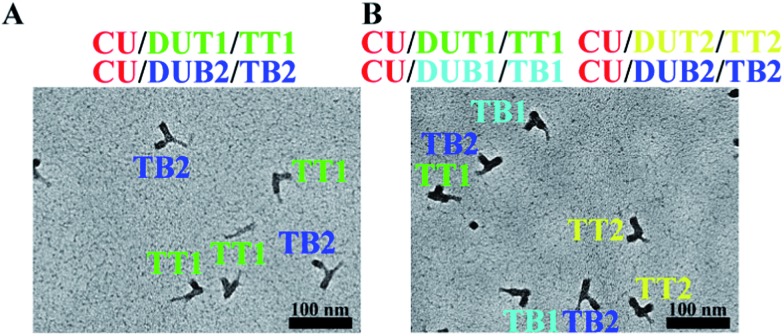
Two-target and four-target DNA detection with PED-SADNA. (A) TEM image of CU, DUT1, and DUB2 in the presence of targets TT1 and TB2. The hybridization was performed separately for CU/DUT1 and CU/DUB2, followed by mixing the two solutions for TEM imaging. (B) TEM image of CU, DUT1, DUT2, DUB1, and DUB2 in the presence of targets TT1, TT2, TB1, and TB2. The hybridization was performed separately for CU/DUT1, CU/DUT2, CU/DUB1 and CU/DUB2, and then the hybridization solutions were combined into a single sample for TEM imaging.

With the single-target assay system validated, a two-target (TT1, TB2) sample was next examined. Accordingly, besides CU and DUT1, a second set of DU (DUB2) was fabricated and purified. The ability of TB2 to bind DUB2 to the target site of CU was then confirmed. For a two-target sample, the hybridization can be performed either separately with CU/DUT1 and CU/DUB2 (with a total ratio of CU/DUT1/DUB2 approximately 2 : 1 : 1; mixing before TEM imaging) or simultaneously with CU/DUT1/DUB2 (with a ratio of approximately 1 : 1 : 1). As expected, separate hybridization gives discrete CU–DUT1 and CU–DUB2 hybrids whereas simultaneous hybridization enables the formation of a CU–DUT1–DUB2 hybrid. In either case, DUT1 and DUB2 hybridize orthogonally with CU in high efficiency only in response to the presence of corresponding targets, TT1 and TB2 (Fig. 3A and S24†), thus demonstrating the high selectivity of the assay system. Although the designed CU system allows for the encoding of four targets, multiplexed interrogation of four targets is a demanding assay scenario because of its contingency upon the elimination of cross-hybridization. Satisfactorily, with the fabrication of two additional sets of DU (DUT2 and DUB1), four targets (TT1, TT2, TB1, TB2) can be unambiguously identified at the expected sites through high-yielding hybridization (Fig. 3B and S29†).

**Fig. 4:**
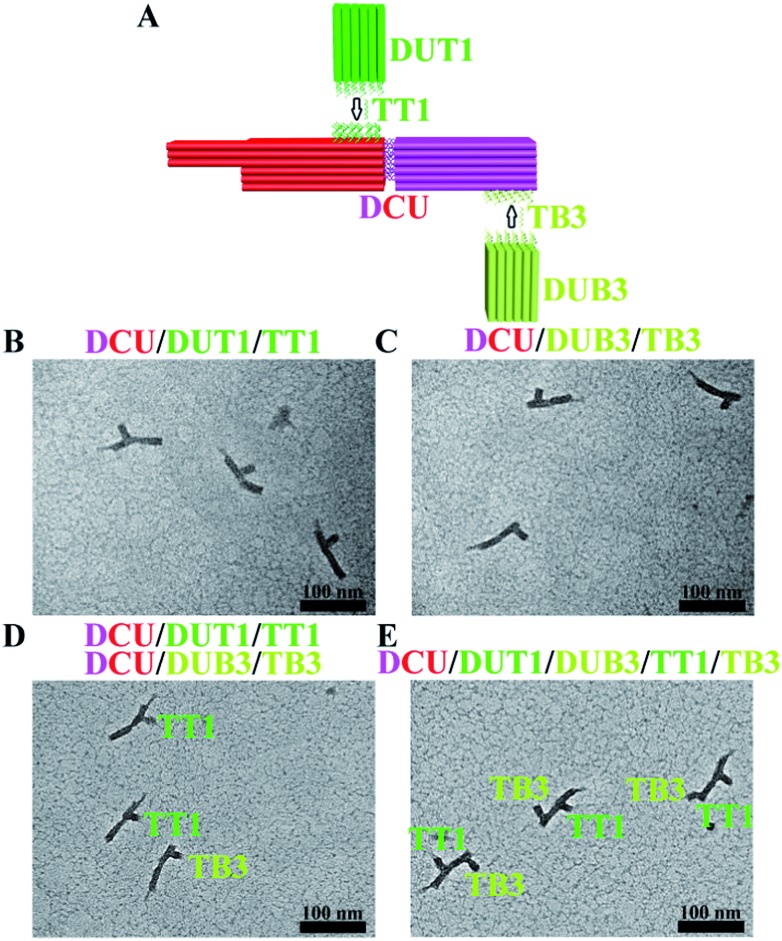
Expansion of positional encoding capacity through an increase in longitudinal dimension. (A) Schematic illustration of DCU design and two-target identification at discrete locations of both cuboids. (B) TEM image of DCU and DUT1 in the presence of target DNA TT1. (C) TEM image of DCU and DUB3 in the presence of target DNA TB3. (D) TEM image of DCU, DUT1, and DUB3 in the presence of targets TT1 and TB3 (hybridization was performed separately for DCU and DU for each target and then the hybridization solutions were combined into a single sample for TEM imaging). (E) TEM image of DCU, DUT1, and DUB3 in the presence of targets TT1 and TB3 (hybridization was performed simultaneously for DCU and DU for two targets and then the sample was subjected to TEM imaging).

In principle, the positional encoding capacity is dictated by the longitudinal dimension of CU as well as the number of CU surfaces that can be distinguished (maximum four surfaces for a cuboid design with a properly configured registry marker). As a proof-of-concept demonstration of the ability to increase longitudinal dimension, a separate cuboid with an identical size to that of the core cuboid part of CU was fabricated and allowed to hybridize with CU to form a double-sized DCU (∼86% yield at a Mg^2+^ concentration of 40 mM) (Fig. 4A). As expected, DCU allows positional encoding at discrete locations of both cuboids (Fig. 4B and C). A test with a two-target (TT1, TB3) sample confirmed orthogonal hybridization and therefore effectiveness of this DCU/DUT1/DUB3 system (Fig. 4D). In addition, the large separation between the two encoding sites enables the better spatially resolved staining and imaging of both DUT1 and DUB3 on one DCU in the case of simultaneous hybridization (Fig. 4E).

## Conclusions

In summary, a multiplexed DNA detection strategy based on PED-SADNA has been developed. This strategy allows the simultaneous achievement of facile positional encoding/decoding and fast hybridization kinetics in a solution assay format. Miniaturized implementation in an ultra-small volume format should enable the routine application of the detection system demonstrated herein in PCR-free settings. Extension of the strategy to the assay of other structurally distinct targets is also foreseeable because of the synthetic availability of a large repertoire of DNA conjugates.
