# Supplementary material for: Multiplexed DNA detection based on positional encoding/decoding with self-assembled DNA nanostructures
Source: Chem Sci. 2014 Oct 15;6(2):930–4. doi: 10.1039/c4sc02696a (PMC5811145; doi:10.1039/c4sc02696a)
Supplement: Supplementary file 1 [file SC-006-C4SC02696A-s001.pdf]

Electronic Supplementary Material (ESI) for Chemical Science.
This journal is © The Royal Society of Chemistry 2014

Supporting Information for

Multiplexed DNA Detection Based on Positional Encoding/Decoding with Self-Assembled DNA Nanostructures

Sha Sun, Huaxin Yao, Feifei Zhang and Jin Zhu*

Department of Polymer Science and Engineering, School of Chemistry and Chemical Engineering, State Key Laboratory of Coordination Chemistry, Nanjing National Laboratory of Microstructures, Nanjing University, Nanjing 210093, China

*Corresponding author. Phone: +86-25-83686291; Fax: +86-25-83317761; Email: jinz@nju.edu.cn

Table of Contents

1. Materials S3
2. Design of Self-Assembled DNA Nanostructures S3-S5
3. Experimental Procedures. S5-S6
4. Screening of $MgCl_2$ Concentration for DNA Hybridization S6-S8
5. Screening of DNA Hybridization Time S8-S10
6. Single-Target DNA Detection S10
7. DNA Detection Limit S11
8. PCU DNA Detection System S12
9. Detection of RNA S13
10. Two-Target DNA Detection S14-S18
11. Four-Target DNA Detection S18-S20
12. Assembly of DCU S21
13. Single-Target and Two-Target DNA Detection by DCU Detection System S22-S26
14. DNA Sequence Information S26-S39
15. References S39

1. Materials

All DNA stands were purchased from Sangon Biotech (Shanghai) Co. Ltd and DL2000 DNA molecular weight marker was from TaKaRa Biotechnology (Dalian) Co. Ltd. Freeze 'N Squeeze column was purchased from Bio-Rad Laboratories, Inc. and uranyl formate was from Polysciences, Inc. Gel electrophoresis was performed on a Bio-Rad system using 2% agarose gel. UV-vis absorption values were obtained using a Eppendorf Biophotometer Plus facility. Transmission electron microscopy (TEM) imaging was performed using a JEOL JEM-1011 facility.

2. Design of Self-Assembled DNA Nanostructures

DNA sequences for the nanostructures were generated by program Sequin.[S1] To reduce undesired interaction patterns, criton size is set to 7, which means any continuous sequence of 7 or more nucleotides (nt) appears at most once.

DNA nanostructures were designed by following Peng Yin's LEGO-like model (for all the DNA sequence information, please see Section 14 of the Supporting Information).[S2,S3] In our design of the core cuboid part of **CU**, DNA double helices were arranged as square lattice bundles, as shown in Figure S1. First, we used Sequin to generate sequences for 36 DNA double helices, each of which is 128 BP (base pairs) in length. Then a sequence of 8Ts (eight continuous thymidines) were added to both ends of one strand of each helix (5'-3' strand for odd helices, 3'-5' strand for even helices, refer to Figure S1 for the odd and even numbering of helices) to prevent non-specific blunt-end stacking. Second, a nick site was created for each helix every 16 nt. Because of the different numbers of nt for the two strands of each helix, the nick sites are staggered by 8 nt. Third, the nick sites of each odd helix were linked with the corresponding sites of neighboring even helix clockwise. Take H9 (helix 9) as an example, its protruding 8Ts on the 5' end is linked to the protruding 8Ts on the 3' end of H14, then the first nick site of H9 should be linked to the first one of H10, the second site of H9 to the second one of H2, the third site of H9 to the third one of H8, and the fourth site of H9 to the fourth one of H14. After the linkage, the remaining 16 nt strands should be merged to 32 nt ones to improve the stability of the nanostructures. The registry marker of **CU** is direct extension of the 9 helices, each of which is 64 BP in length, at one corner of the core cuboid.

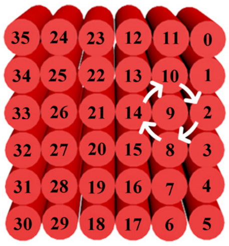

Figure S1. The design of **CU** viewed from the cross-section of the end of core cuboid opposite to the registry marker. This view gives a top left corner location for the registry marker.

The locations of 14 capture probe strands at the top and bottom surfaces of **CU** are shown in Figure S2. The locations of 15 detection probe strands of **DU** are shown in Figure S3 and S4.

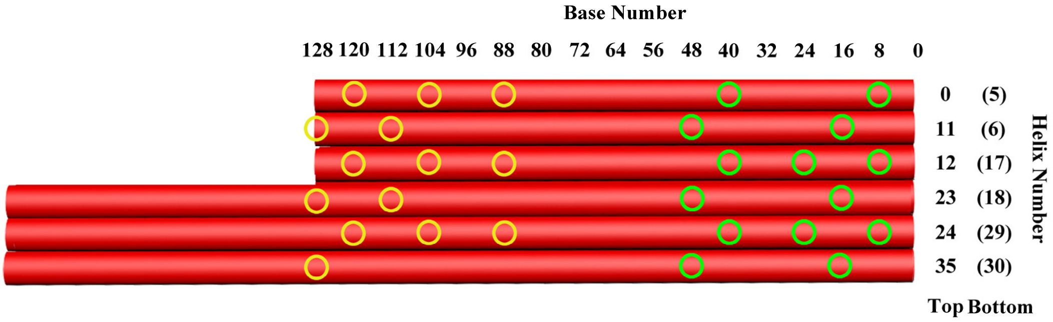

Figure S2. The locations of 14 capture probe strands at the top and bottom surfaces of **CU**, with base numbers and helix numbers specified (circle-marked sites at right: for **TT1** and **TB1**; circle-marked sites at left: for **TT2** and **TB2**).

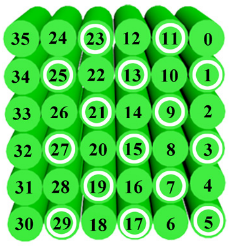

Figure S3. The locations of 15 detection probe strands at **DUT1** (identical for **DUT2**), with the base number (64 BP) and helix numbers (circle-marked sites) specified.

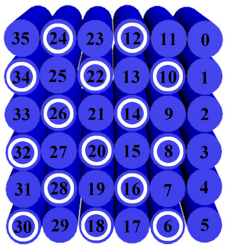

Figure S4. The locations of 15 detection probe strands at **DUB2** (identical for **DUB1**), with the base number (64 BP) and helix numbers (circle-marked sites) specified.

DCU is assembled by **CU** and a cuboid. There are 10 linker strands at the 5' end of **CU** (linker 1: H0, H2, H4, H8, and H10; linker 2: H24, H26, H28, H32, and H34) and they are complementary to the other 10 strands dangling from the 3' end of the cuboid (linker 1: H1, H3, H5, H7, and H9; linker 2: H25, H27, H29, H31, and H33). The locations of 10 linker strands are shown in Figures S5 and S6.

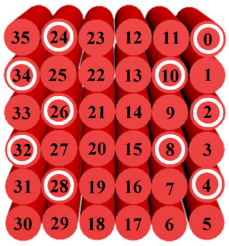

Figure S5. The locations of 10 linker strands at **CU**, with the base number (1 BP) and helix numbers (circle-marked sites) specified.

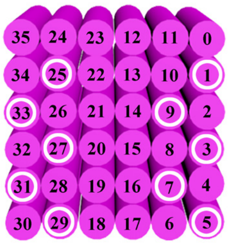

Figure S6. The locations of 10 linker strands at cuboid, with the base number (128 BP) and helix numbers (circle-marked sites) specified.

3. Experimental Procedures

Preparation of nanostructures: Hundreds of single-strand DNA (ssDNA) were mixed and then freeze-dried. After the dissolution of ssDNA in 0.5×TE buffer (5 mM Tris, 1 mM EDTA, pH=8.0, supplemented with 40 mM $MgCl_2$) to a final concentration of 200 nM per strand, the solution was annealed from 90 ^{o}C to 60 ^{o}C at a cooling rate of 5 min/^{o}C and from 60 ^{o}C to 24 ^{o}C at a rate of 2 h/^{o}C.

Purification of nanostructures: The annealed samples were loaded to a native 2% agarose gel with 0.5 μg/mL ethidium bromide (running buffer: 0.5×TBE buffer, containing 44.5 mM Tris, 44.5 mM boric acid, and 1 mM EDTA, supplemented with 11 mM $MgCl_2$) and gel electrophoresis was performed at 80 volts for 2 h in an ice bath. Target bands were excised and cut into small pieces. The gel pieces were placed into Freeze 'N Squeeze columns, frozen at -20 ^{o}C for 5 min and then centrifuged at 7000g at 4 ^{o}C for 5 min.

Hybridization assay: DNA Nanostructures were quantified by measurements of UV-vis absorption values at 260 nm. Different DNA nanostructures were mixed in a molar ratio of 1:1 and target DNA (3 μL) was then added to the mixture. The solution was diluted with 0.5×TBE buffer to a final volume of 10 μL (5 nM for the final concentration of each nanostructure). The hybridization was allowed to proceed at 30 ^{o}C.

TEM imaging: A 2.1 μL of hybridization solution was mixed with 0.3 μL of ssDNA (with a sequence of 5'-GCCTGAAGTCTGGTGCTTAGGCCTTGAAATCA -3' for the generation of a hydrophilic TEM grid surface) and the whole solution was loaded onto a glass slide. On top of the solution was covered with a carbon-coated TEM grid and the contact between the solution and grid was allowed to proceed for 2 min. The TEM grid was then stained with a 2% uranyl formate aqueous solution (containing 25 mM NaOH) for 2 min followed by twice wash with water. TEM imaging was performed at 100 kV.

4. Screening of $MgCl_2$ Concentration for DNA Hybridization

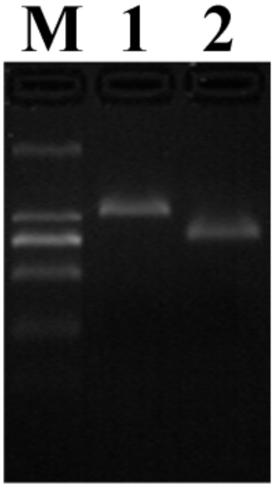

Figure S7. Gel electrophoresis bands for purified **CU** and **DUT1**. Lane M: DL2000 molecular weight marker (same for all the following gel electrophoresis images); Lane 1: **CU**; Lane 2: **DUT1**.

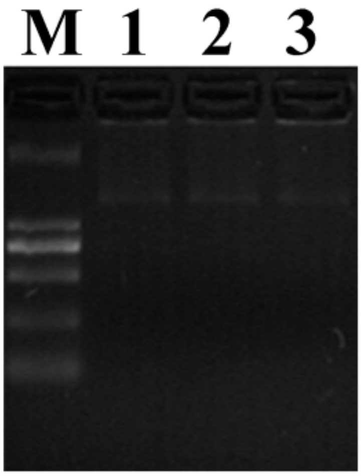

Figure S8. Gel electrophoresis bands for **CU**, **DUT1**, and **TT1** in the presence of different concentrations of $MgCl_2$. Lane 1: 11 mM $MgCl_2$; Lane 2: 20 mM $MgCl_2$; Lane 3: 30 mM $MgCl_2$. The concentration of **TT1** is 300 nM. The non-penetrating material in the gel is the non-specific aggregation of the hybridization product, which probably appears after storage at 4 ^{o}C and could be reduced by pre-heating the sample at 30 ^{o}C before gel electrophoresis (same for all the following gel electrophoresis images).

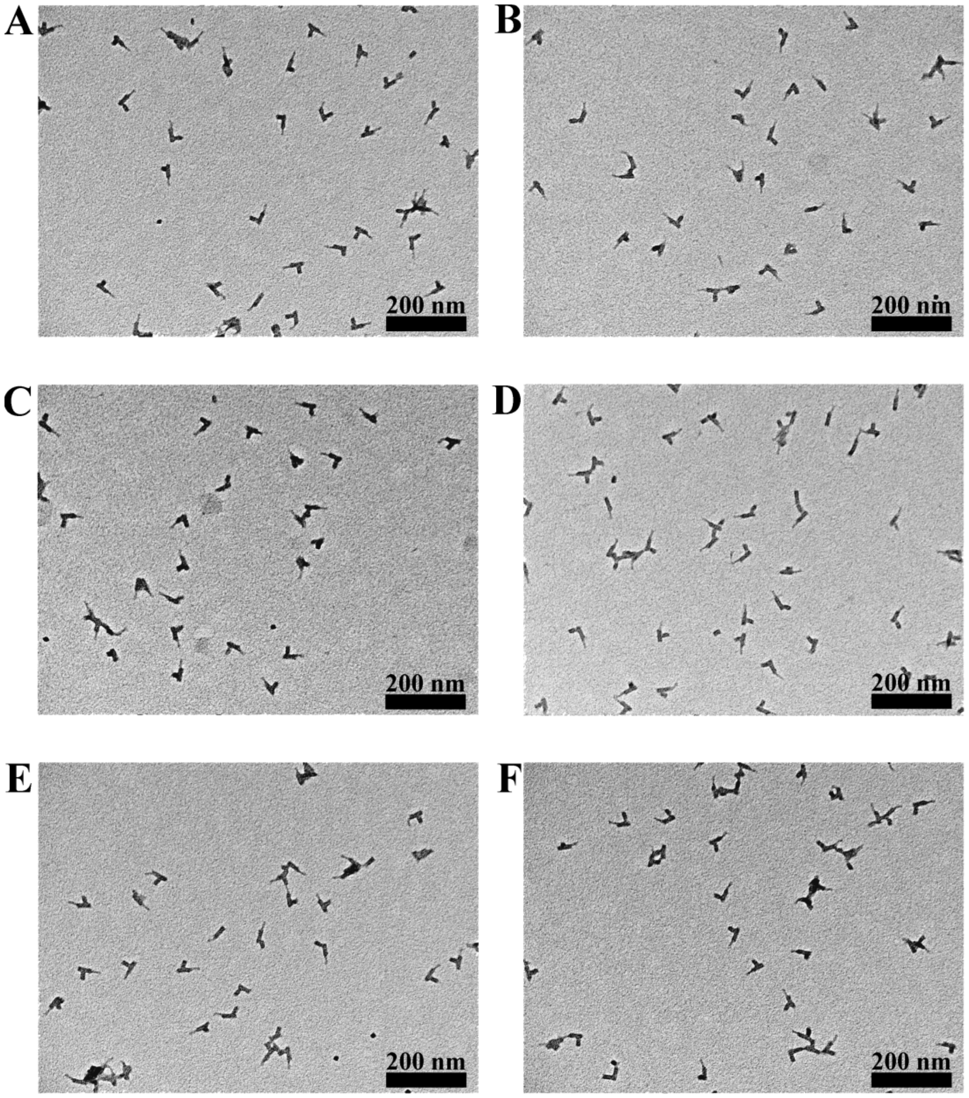

Figure S9. Representative TEM images of **CU**, **DUT1**, and **TT1** in the presence of different concentrations of $MgCl_2$. A) and B): 11 mM $MgCl_2$; C) and D): 20 mM $MgCl_2$; E) and F): 30 mM $MgCl_2$. The concentration of **TT1** is 300 nM.

5. Screening of DNA Hybridization Time

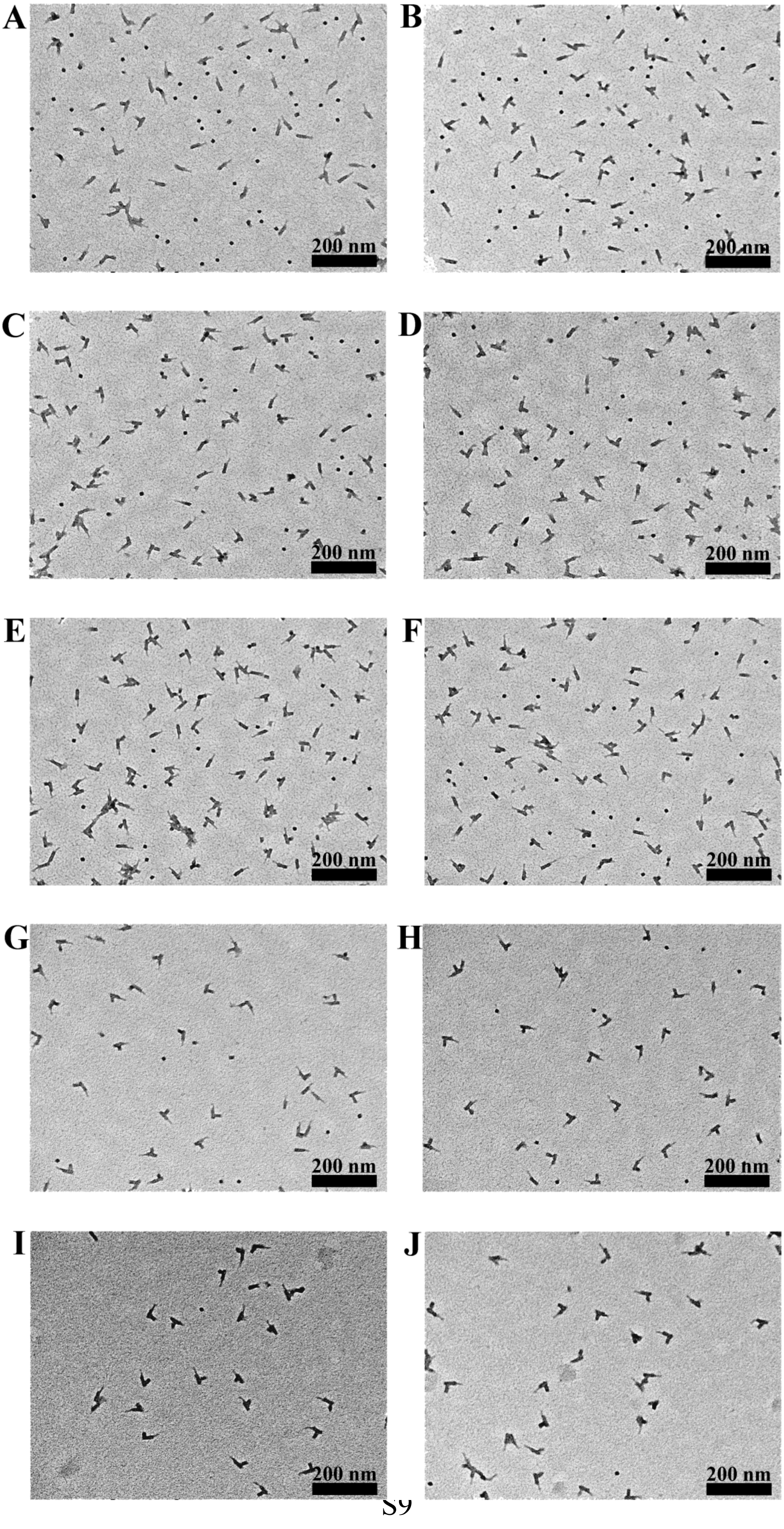
A
B
C
D
E
F
G
H
I
J
200 nm

Figure S10. Representative TEM images of **CU**, **DUT1**, and **TT1** after hybridization for different durations of time. A) and B): 5 min (hybridization percentage, or HP: 41%); C) and D): 10 min (HP: 63%); E) and F): 20 min (HP: 71%); G) and H): 1 h (HP: 88%); I) and J): 8 h (HP: 91%). HP is defined as the percentage of observed **CU**-**DUT1** over all structurally resolved **CU** (calculated from ~300 **CU**). The concentration of **TT1** is 300 nM. The small dark square objects in the images are individual **DUT1** which prefer head-on settlement on the carbon grid instead of side-on settlement as hybridized **DUT1**. All kinds of individual **DU** (**DUT1**, **DUT2**, **DUB1**, **DUB2** and **DUB3)** show head-on settlement in the following TEM images.

6. Single-Target DNA Detection

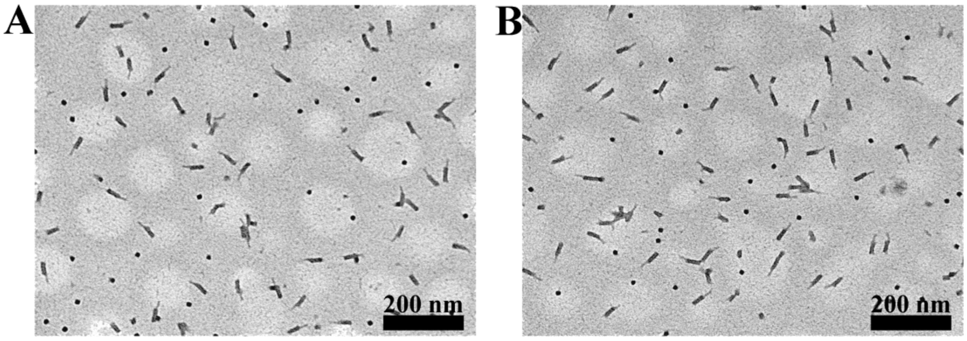

Figure S11. Representative TEM images of **CU** and **DUT1** in the absence of **TT1**.

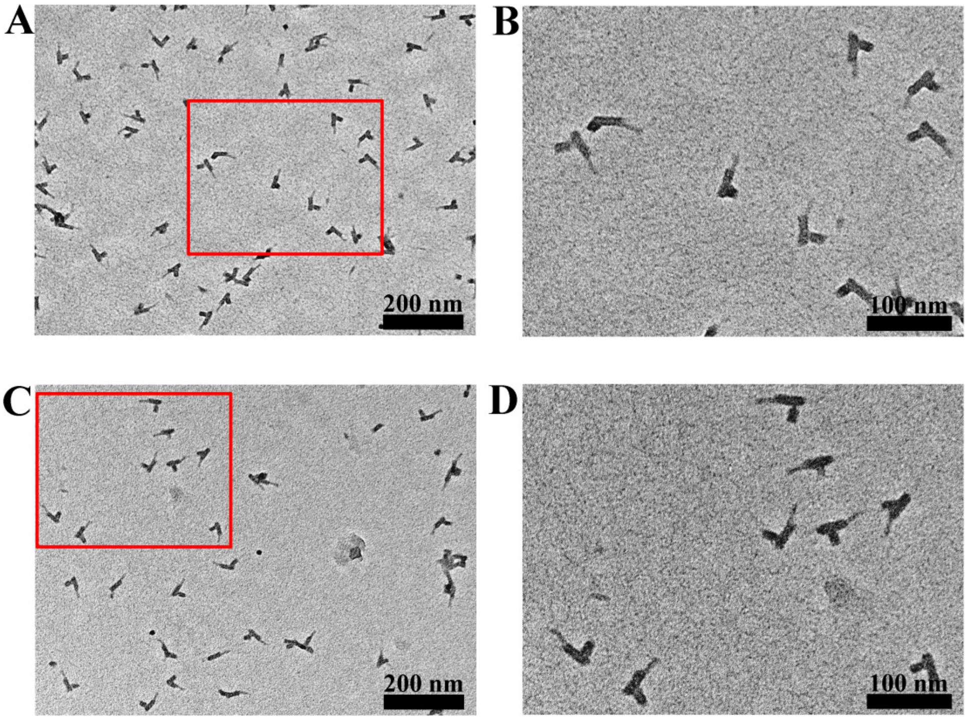

Figure S12. Representative TEM images of **CU** and **DUT1** in the presence of **TT1**. B) and D) are enlarged view images of A) and C), respectively (rectangles marked in red

serve only as an approximate viewing guide for the enlarged area; same for all the following TEM images if applicable). The concentration of **TT1** is 300 nM.

7. DNA Detection Limit

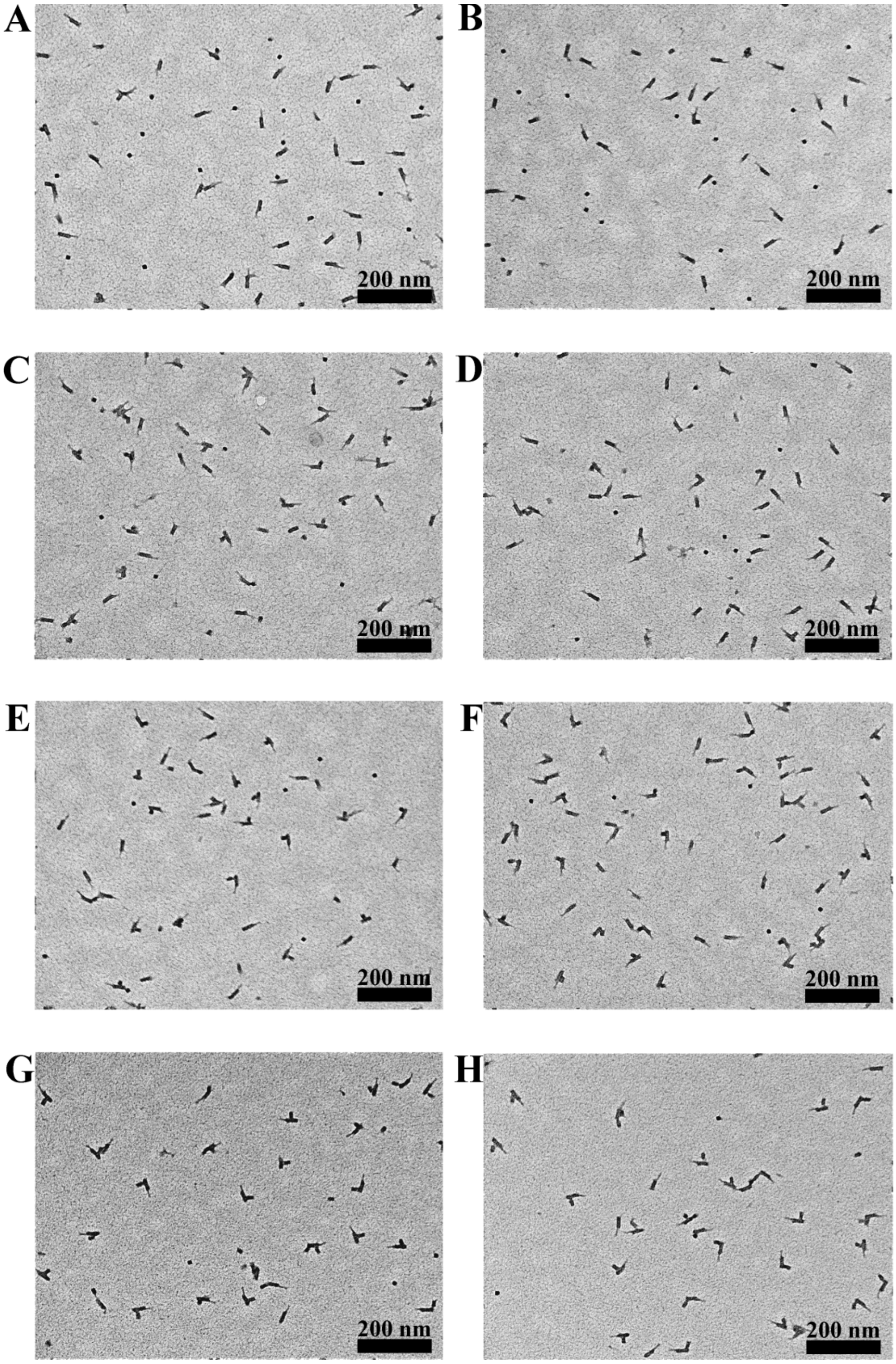

Figure S13. Representative TEM images of **CU** and **DUT1** in the presence of different concentrations of **TT1**. A) and B): 5 nM (HP: 10%); C) and D): 10 nM (HP:

37%); E) and F): 20 nM (HP: 65%). G) and H): 30 nM (HP: 88%). HP was determined by calculation from ~250 **CU**.

8. PCU DNA Detection System

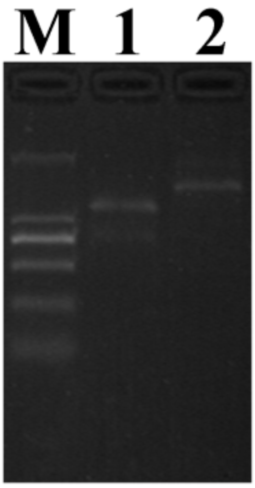

Figure S14. Gel electrophoresis bands for **PCU** and **DUT1** in the absence (lane 1) and presence (lane 2) of **TT1**. The concentration of **TT1** is 300 nM.

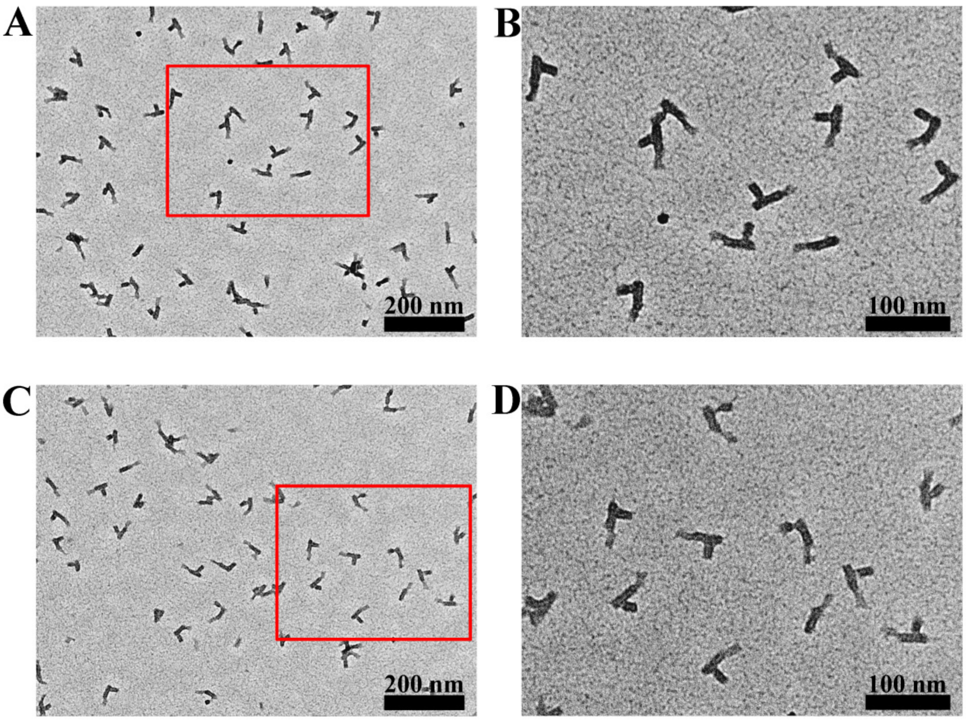

Figure S15. Representative TEM images of **PCU** and **DUT1** in the presence of **TT1**. B) and D) are enlarged view images of A) and C), respectively. The concentration of **TT1** is 300 nM.

9. Detection of RNA

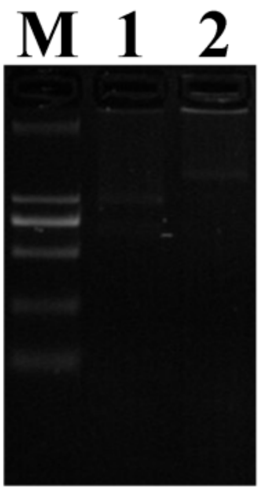

Figure S16. Gel electrophoresis bands for **CU** and **DUT1** in the absence (lane 1) and presence (lane 2) of **RTT1**. The concentration of **RTT1** is 300 nM.

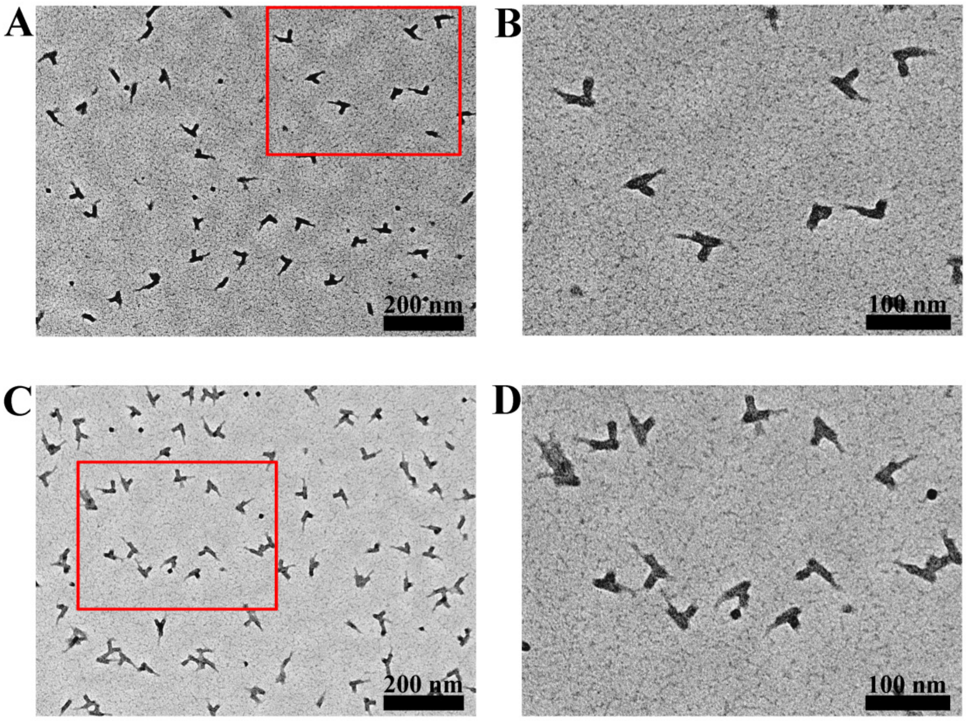

Figure S17. Representative TEM images of **CU** and **DUT1** in the presence of **RTT1**. B) and D) are enlarged view images of A) and C), respectively. The concentration of **RTT1** is 300 nM.

10. Two-Target DNA Detection

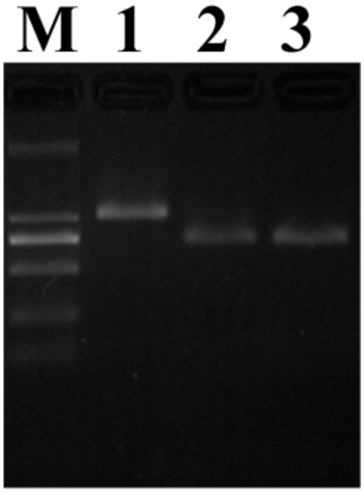

Figure S18. Gel electrophoresis bands for purified **CU**, **DUT1**, and **DUB2**. Lane 1: **CU**; Lane 2: **DUT1**; Lane 3: **DUB2**.

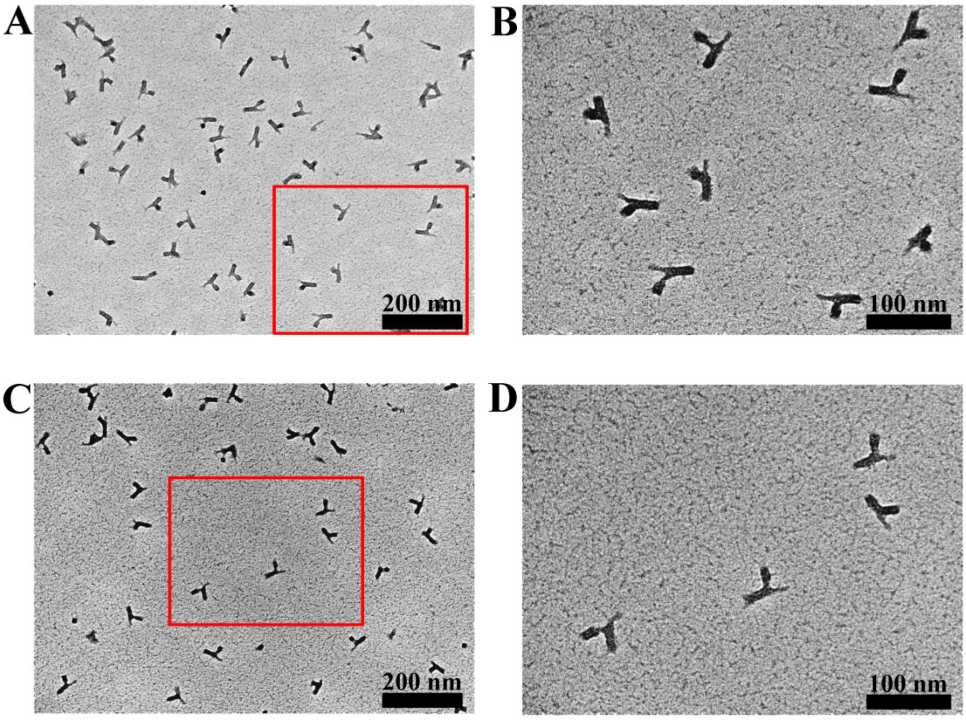

Figure S19. Representative TEM images of **CU** and **DUB2** in the presence of **TB2**. B) and D) are enlarged view images of A) and C), respectively. The concentration of **TB2** is 300 nM.

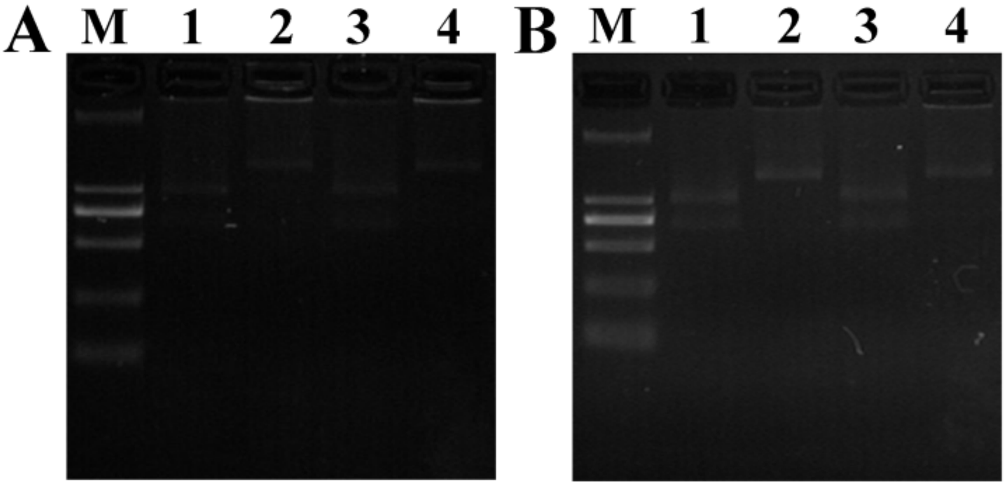

Figure S20. Gel electrophoresis bands for two-target detection (separate hybridization). A) **CU** and **DUT1** in the absence (lane 1) and presence (lane 2) of **TT1**, **CU** and **DUT1** in the presence of **TB2** (lane 3), **CU** and **DUT1** in the presence of both **TT1** and **TB2** (lane 4); B) **CU** and **DUB2** in the absence (lane 1) and presence (lane 2) of **TB2**, **CU** and **DUB2** in the presence of **TT1** (lane 3), **CU** and **DUB2** in the presence of both **TT1** and **TB2** (lane 4). The concentrations of **TT1** and **TB2** are 300 nM.

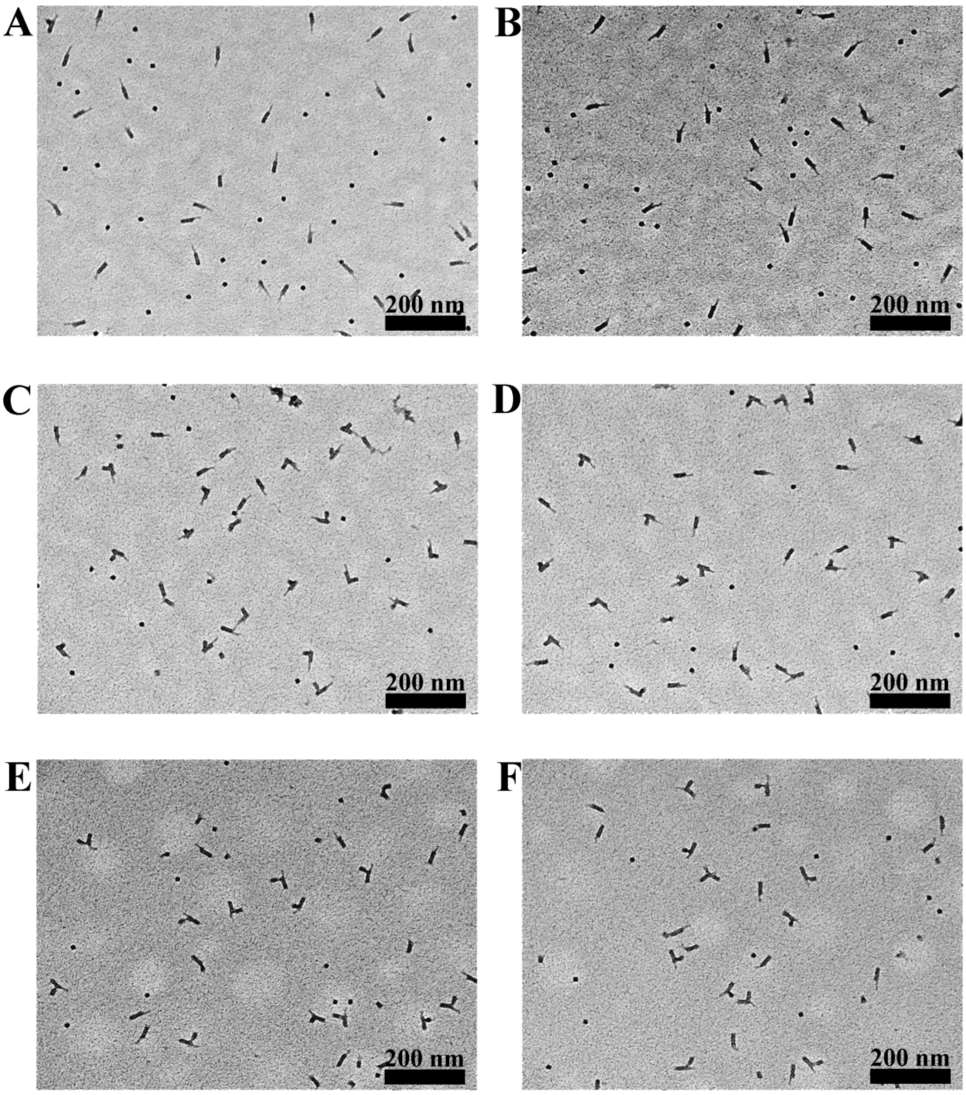

Figure S21. Representative TEM images for two-target detection (in the presence of one target). A) and B) **CU** and **DUT1**, **CU** and **DUB2** in the presence of neither **TT1** nor **TB2**; C) and D) **CU** and **DUT1**, **CU** and **DUB2** in the presence of **TT1** (HP: 50%); E) and F) **CU** and **DUT1**, **CU** and **DUB2** in the presence of **TB2** (HP: 48%). HP was determined by calculation from ~250 **CU**. The concentrations of **TT1** and **TB2** are 300 nM.

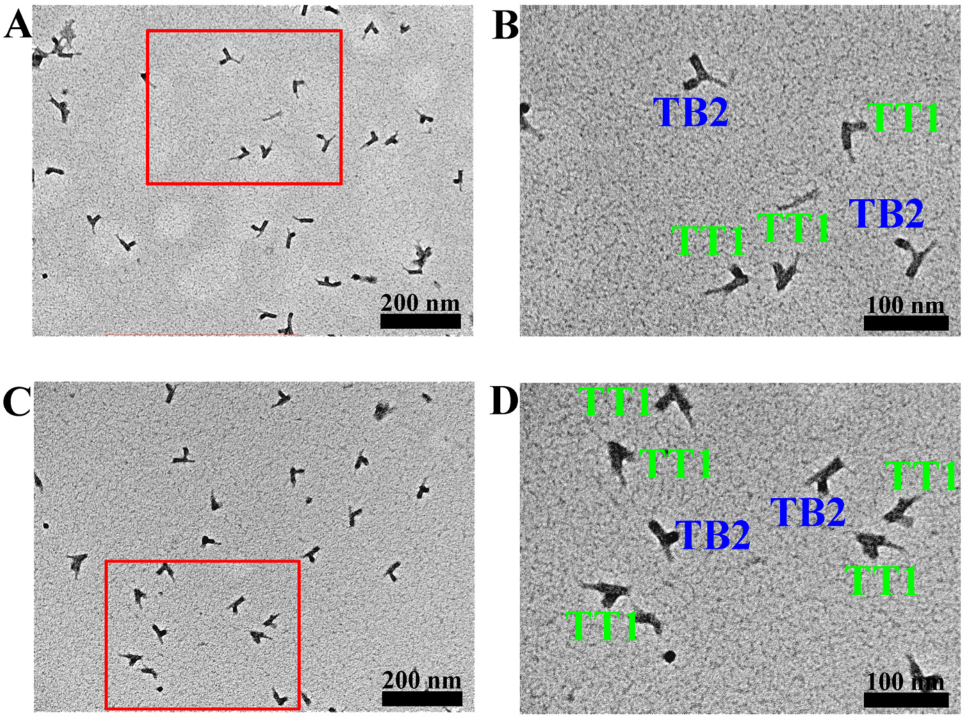

Figure S22. Representative TEM images for two-target detection (separate hybridization, in the presence of two targets). **CU** and **DUT1**, **CU** and **DUB2** in the presence of both **TT1** and **TB2** (HP for **CU-DUT1**: 46%; HP for **CU-DUB2**: 45%). B) and D) are enlarged view images of A) and C), respectively. HP was determined by calculation from ~250 **CU**. The concentrations of **TT1** and **TB2** are 300 nM.

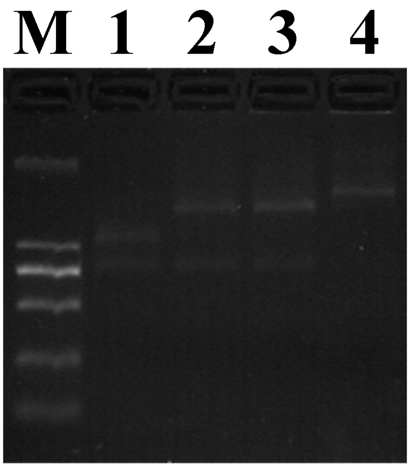

Figure S23. Gel electrophoresis bands for two-target detection (simultaneous hybridization). Lane 1: **CU**, **DUT1**, and **DUB2** in the absence of either **TT1** or **TB2**; Lane 2: **CU**, **DUT1**, and **DUB2** in the presence of **TT1**; Lane 3: **CU**, **DUT1**, and **DUB2** in the presence of **TB1**; Lane 4: **CU**, **DUT1**, and **DUB2** in the presence of both **TT1** and **TB2**. The concentrations of **TT1** and **TB2** are 300 nM.

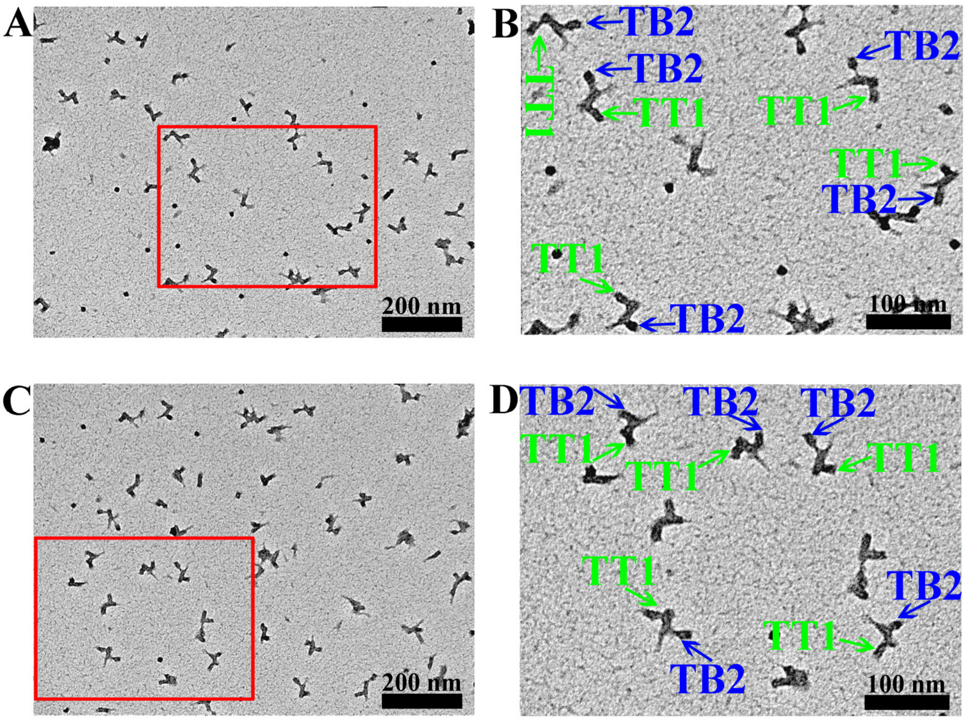

Figure S24. Representative TEM images for two-target detection (simultaneous hybridization). **CU**, **DUT1**, and **DUB2** in the presence of both **TT1** and **TB2** (HP for **CU-DUT1**: 80%; HP for **CU-DUB2**: 83%; for calculation of HP in the case of simultaneous hybridization, **CU-DUT1** and **CU-DUB2** are counted for any structure containing **CU-DUT1** and **CU-DUB2**, respectively). B) and D) are enlarged view images of A) and C), respectively. HP was determined by calculation from ~200 **CU**. The concentrations of **TT1** and **TB2** are 300 nM.

11. Four-Target DNA Detection

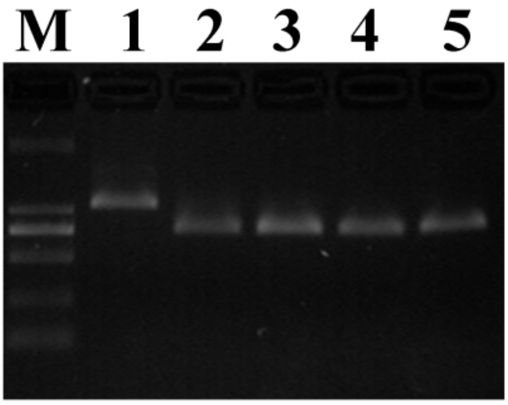

Figure S25. Gel electrophoresis bands for purified **CU**, **DUT1**, **DUT2**, **DUB1**, and **DUB2**. Lane 1: **CU**; Lane 2: **DUT1**; Lane 3: **DUT2**; Lane 4: **DUB1**; Lane 5: **DUB2**.

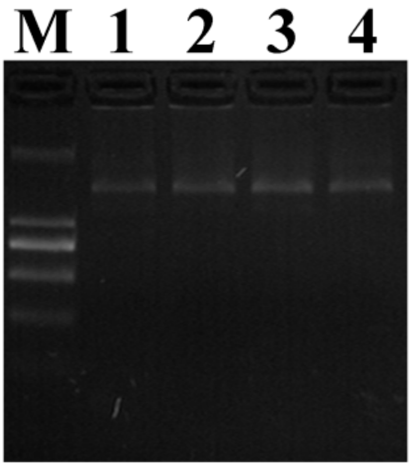

Figure S26. Gel electrophoresis bands for four-target (**TT1**, **TT2**, **TB1**, **TB2**; separate hybridization) detection. Lane 1: **CU** and **DUT1** in the presence of four targets; Lane 2: **CU** and **DUT2** in the presence of four targets; Lane 3: **CU** and **DUB1** in the presence of four targets; Lane 4: **CU** and **DUB2** in the presence of four targets.

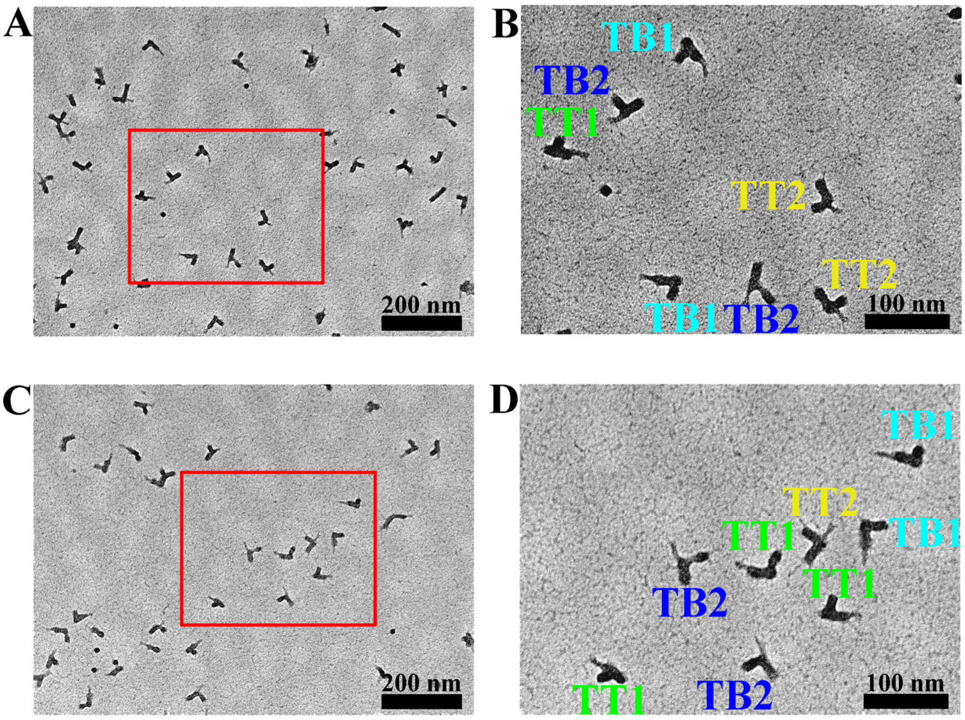

Figure S27. Representative TEM images for four-target detection (separate hybridization). **CU** and **DUT1**, **CU** and **DUT2**, **CU** and **DUB1**, **CU** and **DUB2** in the presence of four targets (**TT1**, **TT2**, **TB1**, **TB2**) (HP for **CU-DUT1**: 22%; HP for **CU-DUT2**: 23%; HP for **CU-DUB1**: 25%; HP for **CU-DUB2**: 24%). B) and D) are enlarged view images of A) and C), respectively. HP was determined by calculation from ~300 **CU**. The concentrations of four targets are all 300 nM.

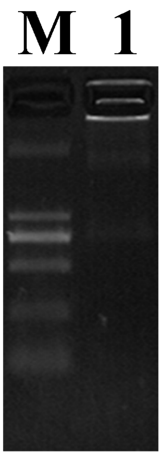

Figure S28. Gel electrophoresis bands for four-target (**TT1**, **TT2**, **TB1**, **TB2**; simultaneous hybridization) detection. Lane 1: **CU**, **DUT1**, **DUT2**, **DUB1**, and **DUB2** in the presence of four targets.

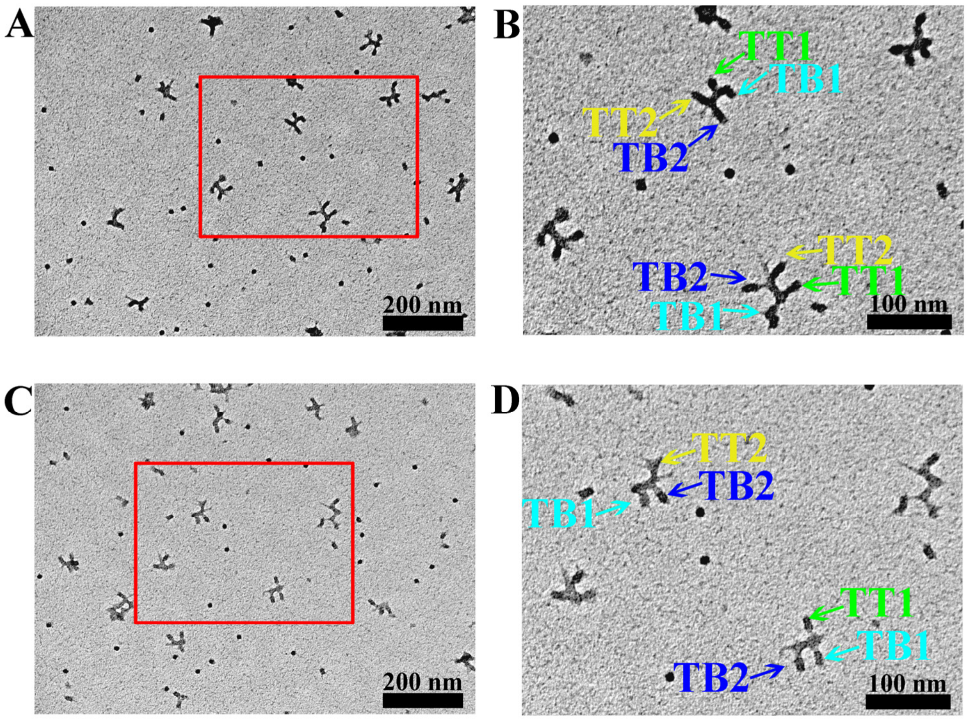

Figure S29. Representative TEM images for four-target detection (simultaneous hybridization). **CU**, **DUT1**, **DUT2**, **DUB1**, and **DUB2** in the presence of four targets (**TT1**, **TT2**, **TB1**, **TB2**) (HP for **CU-DUT1**: 85%; HP for **CU-DUT2**: 75%; HP for **CU-DUB1**: 79%; HP for **CU-DUB2**: 86%). B) and D) are enlarged view images of A) and C), respectively. HP was determined by calculation from ~200 **CU**. The concentrations of four targets are all 300 nM.

12. Assembly of DCU

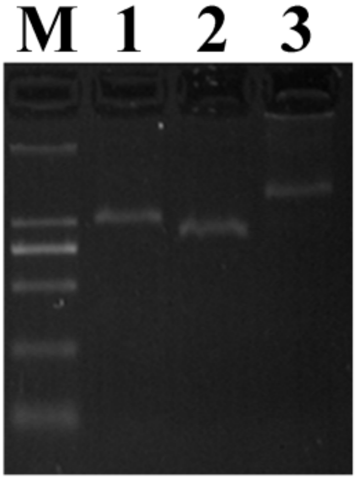

Figure S30. Gel electrophoresis bands for purified **CU**, a purified cuboid with a size identical to the core cuboid part of **CU**, and **DCU**. Lane 1: **CU**; Lane 2: cuboid; Lane 3: **DCU**.

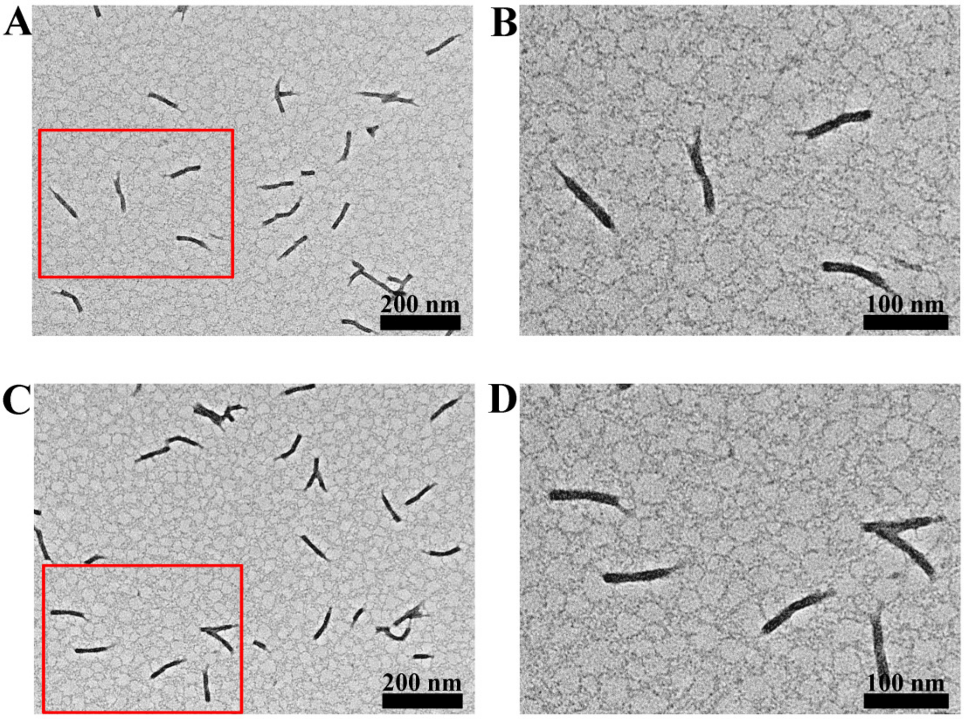

Figure S31. Representative TEM images of **DCU** (HP: 86%). HP is defined as the percentage of observed **DCU** over all structurally resolved **CU** (calculated from ~250 **CU**).

13. Single-Target and Two-Target DNA Detection by DCU Detection System

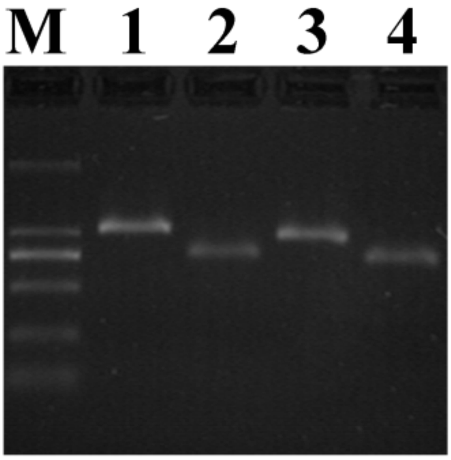

Figure S32. Gel electrophoresis bands for purified **CU**, **DUT1**, cuboid, and **DUB3**. Lane 1: **CU**; Lane 2: **DUT1**; Lane 3: cuboid; Lane 4: **DUB3**.

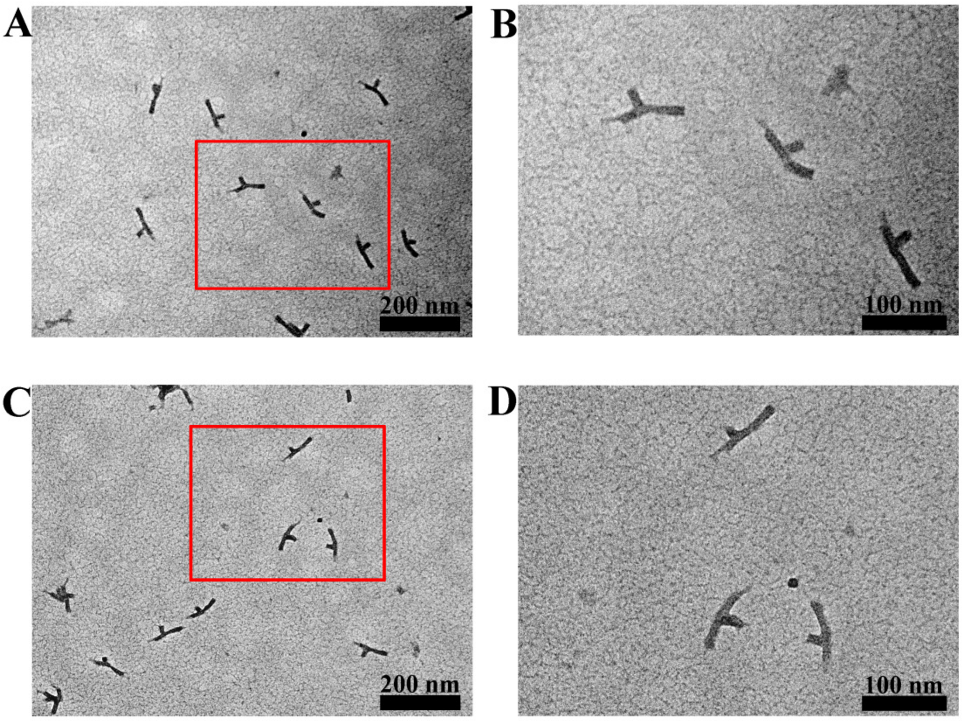

Figure S33. Representative TEM images of **DCU** and **DUT1** in the presence of **TT1** (HP: 92%). B) and D) are enlarged view images of A) and C), respectively. HP is defined as the percentage of observed **DCU-DUT1** over all structurally resolved **DCU** (calculated from ~200 **DCU**). The concentration of **TT1** is 300 nM.

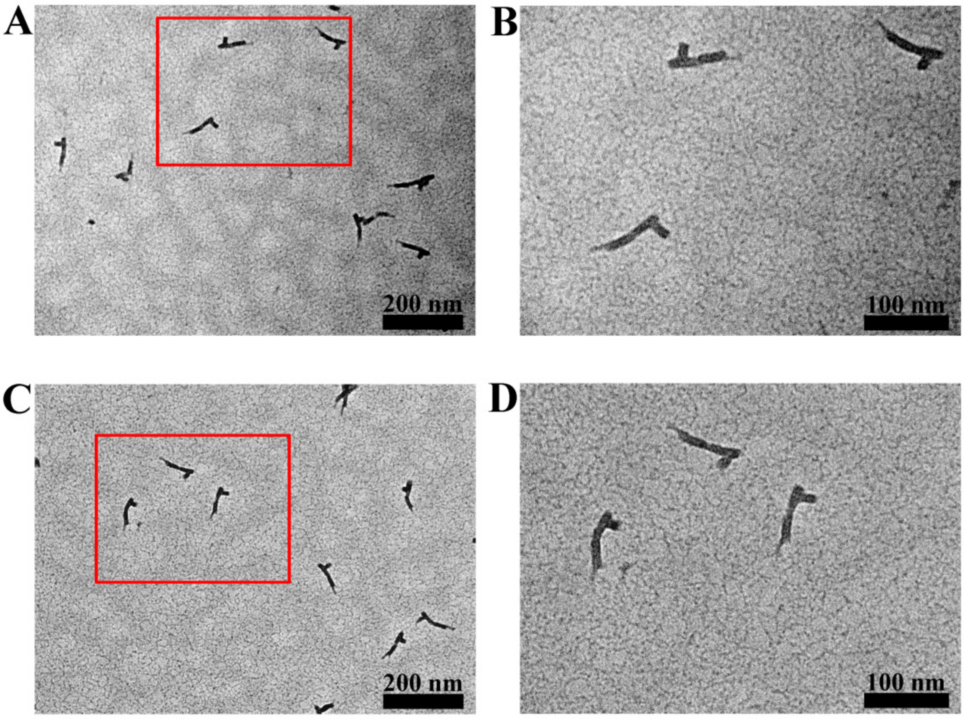

Figure S34. Representative TEM images of **DCU** and **DUB3** in the presence of **TB3** (HP: 93%). B) and D) are enlarged view images of A) and C), respectively. HP is defined as the percentage of observed **DCU-DUB3** over all structurally resolved **DCU** (calculated from ~200 **DCU**). The concentration of **TB3** is 300 nM.

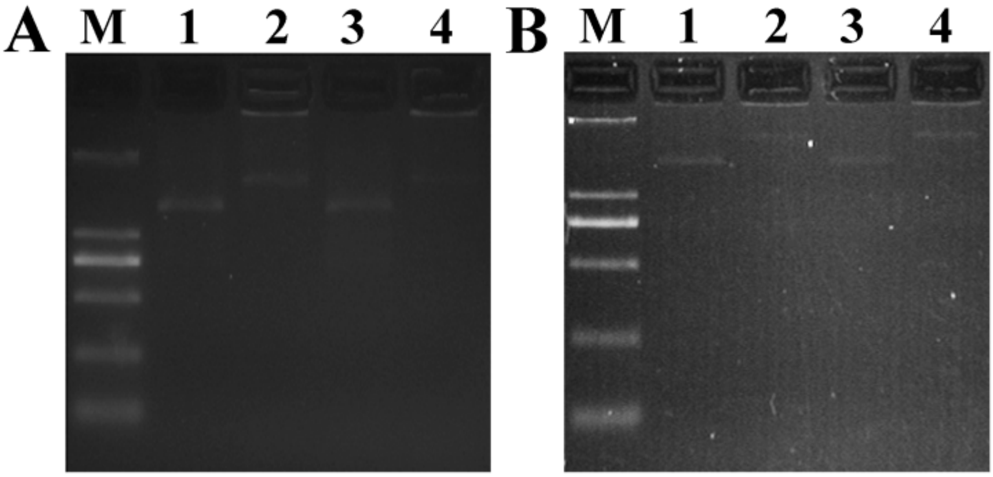

Figure S35. Gel electrophoresis bands for two-target detection. A) **DCU** and **DUT1** in the absence (lane 1) and presence (lane 2) of **TT1**, **DCU** and **DUT1** in the presence of **TB3** (lane 3), **DCU** and **DUT1** in the presence of both **TT1** and **TB3** (lane 4); B) **DCU** and **DUB3** in the absence (lane 1) and presence (lane 2) of **TB3**, **DCU** and **DUB3** in the presence of **TT1** (lane 3), **DCU** and **DUB3** in the presence of both **TT1** and **TB3** (lane 4). The concentrations of **TT1** and **TB3** are 300 nM.

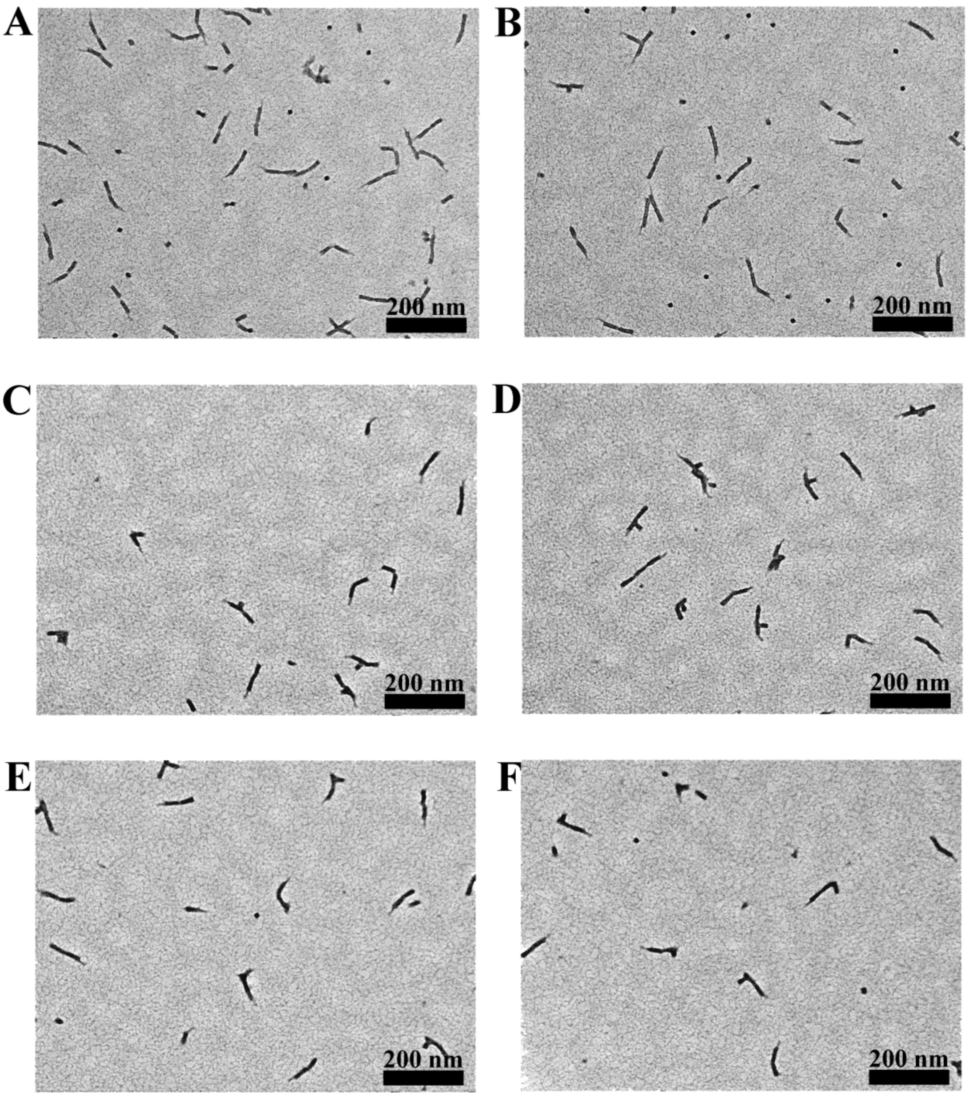

Figure S36. Representative TEM images for two-target detection (in the presence of one target). A) and B) **DCU** and **DUT1**, **DCU** and **DUB3** in the presence of neither **TT1** nor **TB3**; C) and D) **DCU** and **DUT1**, **DCU** and **DUB3** in the presence of **TT1** (HP for **DCU-DUT1**: 47%); E) and F) **DCU** and **DUT1**, **DCU** and **DUB3** in the presence of **TB3** (HP for **DCU-DUB3**: 51%). HP was determined by calculation from ~200 **DCU**. The concentrations of **TT1** and **TB3** are 300 nM.

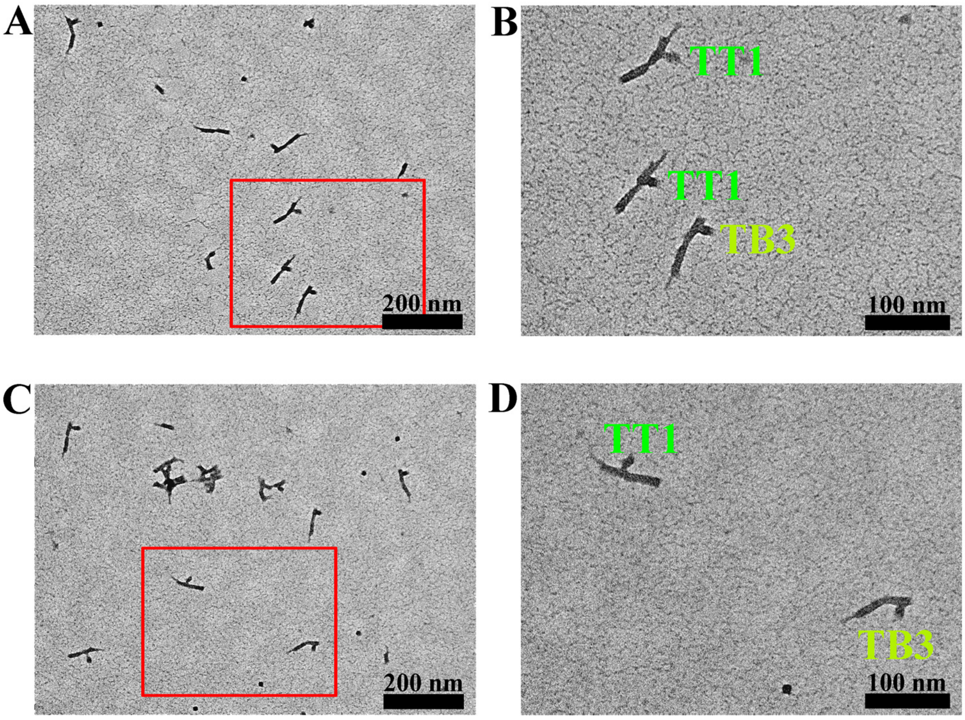

Figure S37. Representative TEM images for two-target detection (separate hybridization, in the presence of two targets). A)-D) **DCU** and **DUT1**, **DCU** and **DUB3** in the presence of both **TT1** and **TB3** (HP for **DCU-DUT1**: 44%; HP for **DCU-DUB3**: 48%). B) and D) are enlarged view images of A) and C), respectively. HP was determined by calculation from ~200 **DCU**. The concentrations of **TT1** and **TB3** are 300 nM.

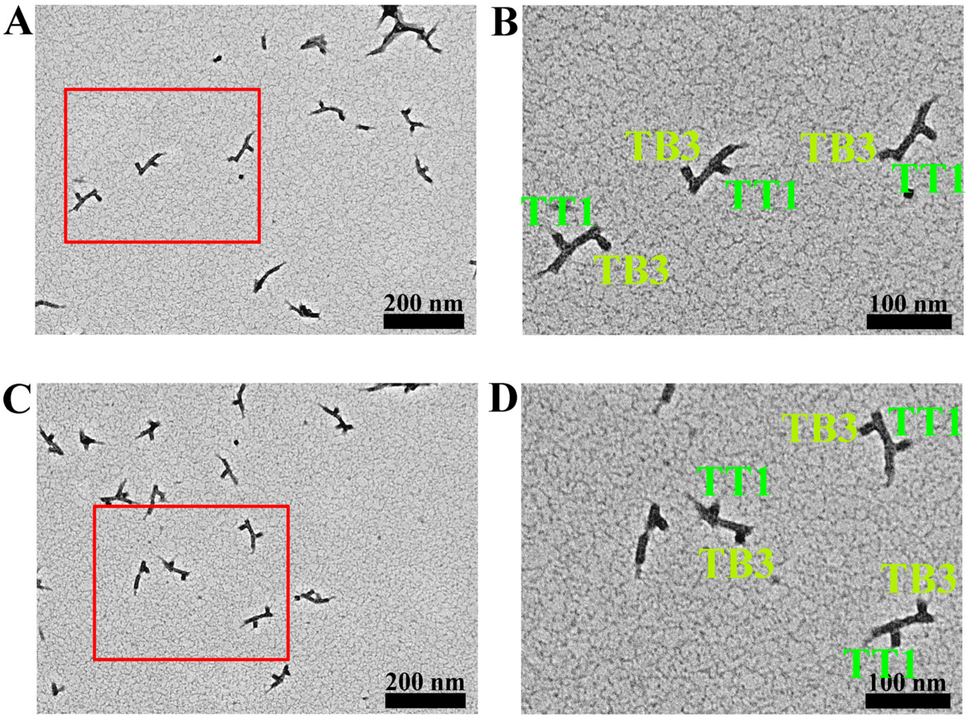

Figure S38. Representative TEM images for two-target detection (simultaneous hybridization). **DCU**, **DUT1**, and **DUB3** in the presence of both **TT1** and **TB3** (HP for **DCU-DUT1-DUB3**: 76%). B) and D) are enlarged view images of A) and C), respectively. HP was determined by calculation from ~200 **DCU**. The concentrations of **TT1** and **TB3** are 300 nM.

14. DNA Sequence Information

Table S1. Probe and target sequences

Name	Sequence (5'-3')	Length
**CU TT1** capture probe	GGCTTCTAAAG	11
**CU TT2** capture probe	AACGGCAGGAA	11
**CU TB1** capture probe	GAATTATGAGT	11
**CU TB2** capture probe	GCAAGGGTCAC	11
**DCU TB3** capture probe	CGATGTGTCGC	11
**DUT1** detection probe	GGTTGTTGGATTTCA	15
**DUT2** detection probe	GGCTGGCAGGATGCT	15
**DUB1** detection probe	GTCGGTTCACGGAGC	15
**DUB2** detection probe	GACCGAGTTACTGTT	15
**DUB3** detection probe	GCAGCTCGTGGACCA	15
**DCU** linker-1 on **CU**	TAAGAGCTATGGG	13
**DCU** linker-2 on **CU**	CAACAGAGGCAGA	13
**DCU** linker-1 on cuboid	CCCATAGCTCTTA	13
**DCU** linker-2 on cuboid	TCTGCCTCTGTTG	13
**TT1**	CTTTAGAAGCCTGAAATCCAACAACC	26

**TT2**	TTCCTGCCGTTAGCATCCTGCCAGCC	26
**TB1**	GCTCCGTGAACCGACACTCATAATTC	26
**TB2**	AACAGTAACTCGGTCGTGACCCTTGC	26
**TB3**	TGGTCCACGAGCTGCGCGACACATCG	26
**RTT1**	CUUUAGAAGCCUGAAAUCCAACAACC	26

Table S2. DNA sequences for the assembly of nanostructures (the sequences used for each nanostructure is marked in the corresponding color: **CU**, **PCU**, **CU for DCU**, **cuboid for DCU**, **DUT1**, **DUT2**, **DUB1**, **DUB2**, **DUB3**)

Name	Sequence (5'-3')	Length
1	CCGCTTCCTTTTTTTTTTTTTTTTGTGCTTGC	32
2	TTGACACCTTTTTTTTTTTTTTTTGGTTCGGA	32
3	ACGCGGCCTTTTTTTTTTTTTTTTGGTGAGGA	32
4	CCGCCTCCTTTTTTTTTTTTTTTTGGAGCAAG	32
5	GCCACGACGCAAGCACGTATTATGGCGAAGAT	32
6	GGCTCAGCTCCGAACCGGAAGGGCTATGATAA	32
7	CATATCCCTCCTCACCGGAAGCGGGAGGATGG	32
8	CATCCGTCTTTGTCCCGGAGGCGGGTGCGAGA	32
9	ATTATCCTATGTCCTCGGCCGCGTGAGTTAGA	32
10	TAACTATTACAATACCGAGATCGAGGTCTTAG	32
11	CTCATTTCTTATCATAGGGATATGGAAACTAG	32
12	GTACCGTCATCTTCGCGGAATTAAACCGGGGT	32
13	AATTAGATCCGGGTCGAGGATAATGGATGTTG	32
14	TACTACCTCCATCCTCGACGGATGAGTATATT	32
15	CCACAGATTCTAACTCAATAGTTAATGTCAGA	32
16	ATCCACGCTCTCGCACTGGCCGGGACGAAGGC	32
17	GATCCCGTATCGTAATGTCGTGGCATTACGAT	32
18	AACCCTTTACCCCGGTAGGTAGTAGTTGAAAG	32
19	TTAAATATCTAGTTTCATCTAATTGGTAGGGT	32
20	ATTTCGTGAATATACTATCTGTGGGGCAGATG	32
21	CCCCGTGCGCCTTCGTGTTGTGAAGTTCGAGC	32
22	AGCCCGTTTCTGACATCGGAATCAACAAGTCA	32
23	TTATTCTTCTTTCAACACGGGATCGGGCACAG	32
24	TGCCGCCCACCCTACCATGTGACAAGGTTTGA	32
25	CGCATCTACATCTGCCATATTTAAGTTTTCAG	32
26	CGTCCGTACCCGTCCAAAAGGGTTGGAGATAG	32
27	GGACTCTAGCTCGAACCACGAAATAGAAAGCA	32
28	ATTAGCCGTGACTTGTGTGCCGACAATGCAGT	32
29	TCCCTGCGCTGTGCCCAAAGGCTGGATAGAGA	32
30	GCTTAGCTTCAAACCTTAGGCGTAGTGTGGTG	32
31	CCTCTACCCTGAAAACAAGAATAAGGTATGGG	32
32	CCAGCACCTGCTTTCTTACGGACGCTGACGTA	32
33	TTACAGCTACTGCATTTAGATGCGGGCGCGAG	32

34	TCGTGCCTTGGTCCCATAGAGTCCAGGGCAAG	32
35	CGCTACAACACCACACGGTAGAGGAAGAGAGG	32
36	TCAACCATTCTCTATCGGGTGATAGTGATTAA	32
37	ATATAACGTACATTGCAGCTGTAAATTGAATG	32
38	CGATTACTCCCATACCGGTGCTGGTGACGAGG	32
39	CGATCATGCTCGCGCCAGGCACGAGAACGTGG	32
40	CTCCTGCCTACGTCAGGCAATAGCAAACGAAT	32
41	AACTCCACTTCTACTCTTGTAGCGAGGCAACG	32
42	CATTTATCTTAATCACAGTAATCGCTGCGAAA	32
43	TACTTCCACCTCTCTTCGTTATATGCGATGTC	32
44	GCACTCGCCCTCGTCACATGATCGCGGAGACA	32
45	CAATATGTATTCGTTTAACATGGGCTCGGGTT	32
46	GTTCAGACCCACGTTCTTGCGGATACAAGAGC	32
47	TCGATCTCTTTTTTTTTTTTTTTTGGGACAAA	32
48	ATTTGCGCTTTTTTTTTTTTTTTTGAGGACAT	32
49	CATAATACTTTTTTTTTTTTTTTTGGCTAGTAGATTGTTTGGAGTCAG	48
50	GCCCTTCCTTTTTTTTTTTTTTTTGGAGACCAAAGAATGTTGAGCAGG	48
51	ATTTAGCCCGGAGCCCTGTGCTCCTTTTTTTTTTTTTTTTGGTATTGT	48
52	TACTGCCCTTTTTTTT	16
53	CGGAACCCTTTTTTTT	16
54	TTAGAACTCCGATACCCGATATCCTTTTTTTTTTTTTTTTGGGTCCGG	48
55	TACTACGTTCCCTCAACGGCTTACGCTCCGCAGTCGTGGCATTACGAT	48
56	CAATTCTCAATATCCATAAATACCACATCCCGGCTGAGCCGAGACGTG	48
57	TGATTCCGCCACAAACGGCTGCAAGTAGCAAGATGCTGAGTGCCGTAT	48
58	TTCACAACCTAAGACCGTAGACGTTAGGCGAAAAGTCGAAAAAAATGA	48
59	CAGCCTTTCCTGTATAATGGCTAAGGGGCCAAAGGATCGTGAGGTCAT	48
60	TACGCCTATTCCAAATGTTCTGAGGGCCGGTGAAATGTGAGGCATAAA	48
61	CACCGCGCGCTTTATACACTTTACTTTTTACAACGGGCTTGGGACCA	48
62	CCACTATTTTCGGTCACCTGAACCTTAAGTCCGCACGGGGAACACGAT	48
63	AAACAATCTACTAGCCGGAGTACAGGTGGCAGGCAGCGGGGATCAGAA	48
64	TATTGCCATACGTCTTATATCTCGCGGTCCCCGGGTTCCGCGGGATGT	48
65	TTAATTCCCTTGCTCCGGGTGAATTGCGGGGCACCCAAGGACAGCGGA	48
66	ACCCCTGATTACCTGTCACCCGTAATCCGCCCGGTGTCAACGACCCGG	48
67	CCCGGCCACCGGACCCGGGGGCCTGGGGCCCGAGGTTTTAGAGGTCCG	48
68	CTGAATACCTATGACCCGCAGCCCTAAGTTCCGCGCAAATGTTTGTGG	48
69	ACGATCCTTTGGCCCCGGCCGGAGATCAAGGGCATAGGGCACTGGGTG	48
70	CCTTAAACTCCTAGACCCCGCCGCTTTTAACTGAGAATTGGGGTCAGT	48
71	TATCACCCCTATCTCCTGGCCGAGGATTTTAGGGTCGCGGGTAGCGTG	48
72	CACGCCCAGCTATCCGTTACCCGGTCTTCTGGGGGCGGCAGCAATGTA	48
73	GCTATTGCATCGTGTTTGGAATGGGATCTGGGAACTAGGGATCCGGTA	48
74	TAACCACAGTCTTTACACTAATCTCTGAGTTCCGGCTAATGATGGCAG	48
75	TTTTTTTTGGTTTGTA	16
76	TTTTTTTTGGAAAACG	16
77	TTTTTTTTGGGATACT	16

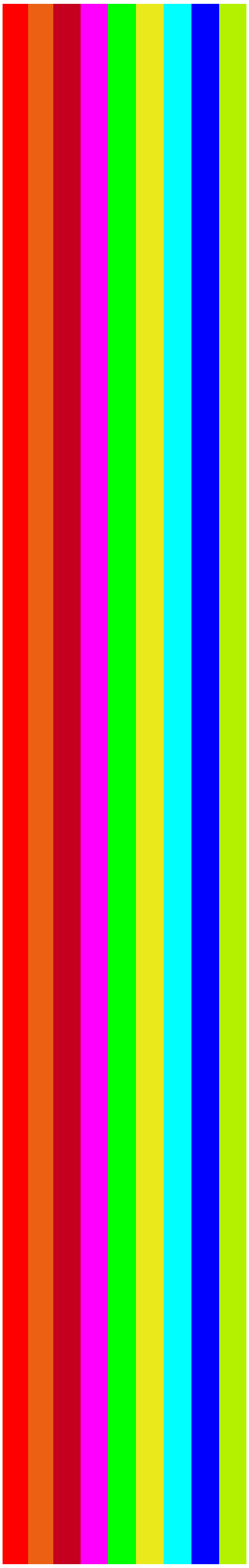

78	ACATTCTTTGGTCTCCGGGCAGTATGCGGAGC	32
79	TTGCAGCCTACAAACCGGAGCACAGGGCTCCG	32
80	ACGTCTACCGTTTTCCGGATATCGGGTATCGG	32
81	TTAGCCATCTGACTCCGTAAGCCGTTGAGGGA	32
82	CTCAGAACCCTGCTCAGGTATTTATGGATATT	32
83	TTCGACTTTTCGCCTAGGCTAAATGTAAAAAA	32
84	TCACATTTCACCGGCCACGTAGTATAGGAAAC	32
85	CATTCGTCATACGGCAGTAAAGTGTATAAAGC	32
86	CTTGACTTTCATTTTTGGTTCAGGTGACCGAA	32
87	TTAACATCAATCACCGCCGGGTAACGGATAGC	32
88	CCGCGACCCTAAAATCGGCAATTGGATACGTA	32
89	CATCTCACAAATCACGAGATTAGTGTAAAGAC	32
90	TATGTCTCATGACCTCATGAGTTGTATGGTAT	32
91	CCAAGTTCTTTATGCCGGGCTGGGAGCCGCGG	32
92	CAAACGCCTTGTGTCTGCGCGGTGTGTATTGA	32
93	CCGTCTCTCGTCTTCCGGGAGATTGGTGGATG	32
94	ATACCCCCTGCCGTCCGAGACGAAAGACAGAG	32
95	TCGTCTATCAACTTCCGGAACGAAGACACGAG	32
96	GCAACCAGCGCGAACCGGTTCGTGCAGAAAGT	32
97	CTGGCCTCCGACCTCCGGAGAGCCGTAAGATG	32
98	CAACTAACTACCATCCGGAACAAGCGTGATAC	32
99	TCCCTGTTCTCTGTCTATAGACGATGACAACG	32
100	TTTCTCATCATCCACCGGGGCTCAATGAGGTA	32
101	TCCACGTCCATTCGCAGAGGCCAGGAATCGTG	32
102	GTCCGCACCTCGTGTCCTGGTTGCTGGGGGTT	32
103	TCTCGGAACATCTTACGTTAGTTGTGTGGGAT	32
104	ATCACCTAACTTTCTGTTACAGGGTGCTAGGT	32
105	TCTGGCTCACCACCCAAACAGGGAAACGAGGA	32
106	ATACTCTATACCTCATGTGCGGACGATAGGTG	32
107	CCACACCCCGTTGTCAGACGTGGAGAATAGAG	32
108	TTTTCACTAACCCCCATTCCGAGAGTCGATGG	32
109	CTATATAAACCTAGCAGAGGCGCGGGGGTCGG	32
110	CCCTGGTCATCCCACAGAAGGGACGCGGTAAA	32
111	TGAACAACCACCTATCGAGCCAGAGGACGGTT	32
112	TACCTGCCCTCTATTCAGTTGGTTGGCGGTCA	32
113	CGATTCGCCCATCGACGGGTGTGGATCGAACC	32
114	ATACCAGCTCTCAGTCTAGAGTATGGAGTGCG	32
115	CTCGACCACCGACCCCAGTGAAAAGATATGTG	32
116	GACTTCCGTTTACCGCGGGACACGTAGAGCGC	32
117	CTTCCATCAACCGTCCAGTGGATAAGGAATAT	32
118	ATAACCTCTGACCGCCACAGTGAGTAGTGAGC	32
119	GCCGTGCCGGTTCGATGTTGTTCAAAGCTGGA	32
120	CCGGTACCCACATATCGCTGGTATGTGTAGTG	32
121	TGCACCTTGCGCTCTAGCGAATCGGGAGCTAA	32

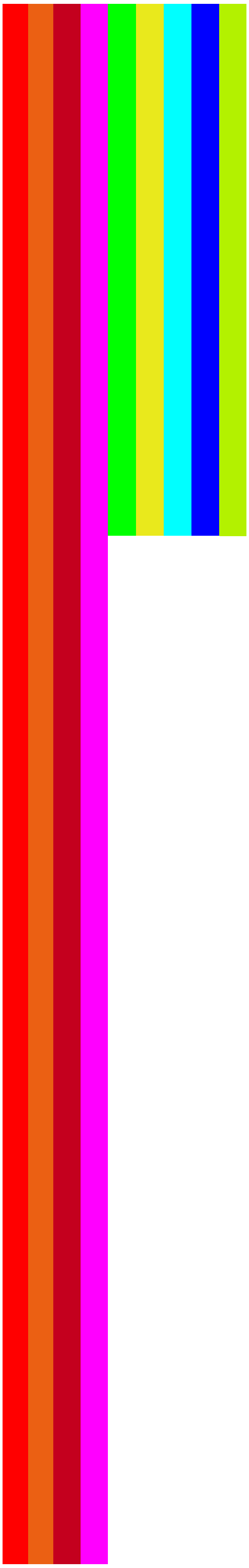

122	CTTATGCTCTTTAGTCTGGTCGAGGGTTATAG	32
123	CGTCCACCGCTCACTAGGCACGGCAAGTGATA	32
124	TGTACCCCATATTCCTTGGTAGATAGCAGTCG	32
125	CCCGTCAACCTTTAGCAAGGTGCAGTGAATGA	32
126	CCTGCGTCTCCAGCTTGGTACCGGGAGACTGG	32
127	CGGTCCTATTAGCTCCAGCATAAGATTTATAA	32
128	CAATCGCGCACTACACAAGGCGACAAGAAGGT	32
129	TCCTGGATTCTTACTGGGTGGACGAGCCGGGT	32
130	TCGCTTCTCGACTGCTGACGCAGGAAGCTGTT	32
131	TGCATTCATATCACTTTGACGGGGGACGTAA	32
132	TGCAATCCCCAGTCTCTAGGACCGAGGGGCGA	32
133	TTCAAGCCTTATAAATAAGGGTGTTCGCATGA	32
134	TTTTTTTTTCATGCGAGTGGGGTGTTTTTTTT	32
135	ACCCGTTAACCTTCTTAATGATAGGCAAGCGG	32
136	TTTTTTTTGCCATCGCAGAAGCGATTTTTTTT	32
137	TTTTTTTTCCGCTTGCGGATTGCATTTTTTTT	32
138	TGGAATCCTCCGTCACGTTTAAGGGGACGTCGGACAACAGAAAAATAA	48
139	TTTTTTTTTTACGTCCGTCAAATCTTTTTTTT	32
140	TTTTTTTTAACAGCTTATCCAGGATTTTTTTT	32
141	TTTTTTTTACCCGGCTGTTTGCGGTTTTTTTT	32
142	TTTTTTTTTAATCTATGGCTTGAATTTTTTTT	32
143	AATCTCCCTCGTTCACGAGACATAGGGACCCGAAGTTTGATAATAGAT	48
144	TTCGTCTCCGTTGCCTGAACTTGGGGAACCAAGAGTCATAAGGGTCTT	48
145	CATTGACCCACGCTACCGAGGTACGGACATTG	32
146	CATGAACCGCAACCTGTGGGCGTGGGTTAAAT	32
147	TGTACACCTACCGGATGCGCGGATGGTGAACA	32
148	CCACGCGCCAGTTTCTTGATTGCCACCCGACGTGTGGTTAGGTTCACG	48
149	TTACTGACACACAGCACACGGGCCTCAATACAGTCTGAACGGATGGTA	48
150	GCGTCGCATTGCACTGTGACCTTCACTTCGTAACATATTGGGCCCGGG	48
151	CGTATTCCCACCCAGTGCCCTATGCCCTTGATGGGGCGCTGCAGGATG	48
152	CGACACCCATACCATACAACTCATGTTTCCTACGCAGGGAGAGTAGAA	48
153	ATGCATCCCCGCGGCTCCCAGCCCACTGACCCAGCTAAGCCAGGTCAG	48
154	ATCCGCAACTGCCATCGACGAATGACGTGTGGCACGCGAAGACAGTTG	48
155	CCCATGTTCTTGCCCTAAGTCAAGAGACACAAGGCGTTTGGGTCGACG	48
156	CCCTAGTTCCCAGATCTTGCAAGCTACGGGCGATCGGAAGGAGTTGCA	48
157	GTCTTGGCCTGACCTGGATGTTAAGTGACGGAGGATTCCAGGTGTCTA	48
158	CCAGCGACCAATGTCCGTACCTCGCATCCTGCATGGTTGAGTGAACGA	48
159	TGTGTCCACATTCAATGTGAGATGCAGGTTGCGGTTCATGATGCACAG	48
160	TCGTTCTATGTTCACCATCCGCGCTACGTATCGGCAGGAGCCAACGGT	48
161	TTCGCGTGCCACACGTACGCATGGCGTCGGGTGGCAATCAAGAAACTG	48
162	CTGTTAACTGCAACTCCTTCCGATCGCCCGTAAATAGTGGTACGAAGT	48
163	TTTTTTTTTCGCCCCTTGAATGCATTTTTTTT	32
164	TTCGTTCCTTTCGCAGGTGGAGTTGGAAGACG	32
165	CACTGTCCGACATCGCGCCAAGACGGACGGCA	32

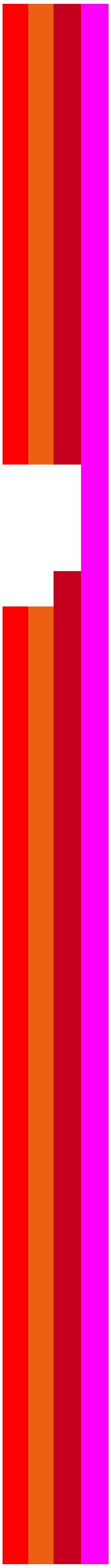

166	GGCTCTCCTGTCTCCGTGGAAGTAGGAAGTTG	32
167	CACGAACCACCGTTGGGATAAATGGGAGTGTT	32
168	CTTGTTCCAACCCGAGGCGAGTGCGGTTCGCG	32
169	AGTTCTCCGCTCTTGTTGGACACAGGAGGTCG	32
170	CACAAGCCACATGACTTCTGTCAAACGTCACAAGAGACGGTGGGTGGT	48
171	GTATCTCAAAACACTATATTGCTCATCGCGTCGGGGGTATTAGGGGAT	48
172	GTCCCTTCTAGCCTTCACTAATGTATATGTTGTGCGGCGTAATATGCT	48
173	CGCGCCTCGTATCACGATCGATGATCTGTAGAACAATGACATGGATCA	48
174	TATCCACTACCCCTTGAATTGGCAGCCGGTCGGCTGATTTAAAGTATA	48
175	CTCACTGTTCCTCGTTGAGGCCTGTCGGTTGTCGAACGATGGTTATTG	48
176	TTTTTTTTGCCTTAATAGCCTGTAGCCCAGACGATGGAAGCAGTAAGA	48
177	TTTTTTTTAGCCGTCAGGTTCCCGTGGCCCGCGAGGTTATGGGTTTAG	48
178	GGTCTTTTGTTCGTCGGCCGCCGGCCGTTTGTGACCAGGGGACTAAAG	48
179	TCGTATAACATTACTTGAGCTACATGCTACACTTATATAGATGGATTA	48
180	TCAAACTTCGGGTCCCGGAATACGGAATTCGGGATTCAGAGCTCAGGG	48
181	TCAACACATTATTTTTCTGTTGTCCGACGTCCGGATGCATGACGCGAT	48
182	TGAGCCCCAACACTCCGGTCAATGTGCAAAAGGCCATAGGCCGCTGGA	48
183	CGCTATTTACTCAGTGCTCTATGCATTTAACCGGACAGTGTGCGAATG	48
184	CCCTGTAACCCGGGCCGGTGTACAGAGTACTGAAATACAGAACGCTGT	48
185	TTGTTATCTGTTTTCTTCGAGACCCGTGAACCGGAGAACTGAAGGCTA	48
186	AAATCAGCCGACCGGCACCTGATGAATAGTAAAATGTCGGATGTCTGG	48
187	ACATCGGTCGCCGATCTTAGTTTATCGTTTATTGAGATACGCGGGCCA	48
188	ATCTACCACGCACTCCCATCAGTGATCAGTAGGCCACTAGGATCCAGT	48
189	CTTCCGCAGTCTACTGTCTATAGTCCGCGTTTGGCAGGTAGCTAAAGG	48
190	GTCGCCTTTAATCCATATGCTAATTGAGTACCAGGATACCTGGTTACT	48
191	GTACCTTGAGTCCTGTTACCGTATACTGTATCCGGAAGTCGGGAAGCC	48
192	TATGACTCTTGGTTCCGGGTGTCGTGTGACGT	32
193	ACATTAGTCAACTGTCGGCCCGTGTGCTGTGT	32
194	TCATCGATCGTCGACCGAAGGTCACAGTGCAA	32
195	TGCCAATTATCTATTATTGACAGAAGTCATGT	32
196	CAGGCCTCAAGACCCTGAGCAATATAGTGTTT	32
197	GTCATTGTTCTACAGAGTCAGTAAACAAACGG	32
198	ATCGTTCGACAACCGAGGCTTGTGGTCTGGGC	32
199	ATACGCCAAGCATATTCCGGCGGCCGACGAAC	32
200	GCAGCCAATGATCCATTGTAGCTCAAGTAATG	32
201	TAATTTATTATACTTTTACAGGCTATTAAGGC	32
202	CCGCAAACCAATAACCCGGGAACCTGACGGCT	32
203	GTGCGTCACGCAGCTAAAAAGACCATAGATTA	32
204	TTTTTTTTACTGGATCAAATTAAGTTTTTTTT	32
205	TTTTTTTTACTCGTCGTGCGGAAGTTTTTTTT	32
206	TTTTTTTTTATATCCTATAAATTATTTTTTTT	32
207	ACACCCTTGGCTTCCCTGGCGTATGTCGATTGACGATTGTTTTTTTTT	48
208	TTTTTTTTACCACGTAACCGATGTTTTTTTTT	32
209	TTTTTTTTCTCTAGTCCAAGGTACTTTTTTTT	32

210	GGCTTCTAAAGCTCCGGCCTTCTGATCCCCGCTGCCTGCCACCGAGCGCTGAGAACCGT	59
211	GGCTTCTAAAGCAGCGCTCCAAGTCCC	27
212	GGCTTCTAAAGAGCGCCCCGTACTACATACATCCTACGGTTCTGACGGTACTATACAGG	59
213	GGCTTCTAAAGATTCACCCTTTTTTTTTTTTTTTTGGGACTTG	43
214	GGCTTCTAAAGCCTTGGGTGCCCCGCAGGACAAGCAGCGTGCG	43
215	GGCTTCTAAAGCTCGGCCATCCGCTGTAGGATGTATGTAGTAC	43
216	GGCTTCTAAAGGCTTGTCCATCGGCCC	27
217	GGCTTCTAAAGCAATTGCCGCTAGCCCCCGAGTCCCGCACGCTGCGTGGATTGGACGGG	59
218	GGCTTCTAAAGTAAAACCTCGGGCCCCGGGCAGCTGGCGGCGAACGGGAGTCCGGCGAT	59
219	GGCTTCTAAAGCCATTCCACGGACCTCGGACTCGGGGGCTAGC	43
220	GGCTTCTAAAGAGCTGCCCAGTATCCC	27
221	GGCTTCTAAAGGCTTGCAAATCGCCGGACTCCCGTTCGCCGCCAGTTCTAAGGACTTAA	59
222	CGAGATATAAGACGTA	16
223	GGCTTCTAAAGAGGCCCCCTTTTTTTTTTTTTTTTGGGCCGAT	43
224	GGCTTCTAAAGTGTACTCCTTTTTTTT	27
225	CCCGCCCCTTTTTTTTTTTTTTTTGGGGACCGGAATTATGAGT	43
226	CCTACCGACTCATAATTGGCAATAAGTTAAAAGCGGCGGGGTCTAGGAGAATTATGAGT	59
227	GGGGCGGGAATCTAGGGAATTATGAGT	27
228	TGTCACATCACGTCTCTGCCAGGGATTATGAGTCGGTAGGCGGTGATTGAATTATGAGT	59
229	CACAGCCCTTTTTTTTTTTTTTTTGGGCGGATGAATTATGAGT	43
230	CCCTGGCACCTAGATTTACGGGTGACAGGTAAGAATTATGAGT	43
231	ACCAGCCACCAGTCACTCAGGGGTCCAGAAGAGAATTATGAGT	43
232	GGGCTGTGGAGCATTGGAATTATGAGT	27
233	GTCGGCACCAACATCCGGATAACGGTGACTGGTGGCTGGTCGTGATTTGAATTATGAGT	59
234	GCGGCCCACAATTTCCAGCATTCCTTTTTTTTTTTTTTTTGGAACTTAGAATTATGAGT	59
235	CGTTATCCCAATGCTCGGGCTGCGGGTCATAGGAATTATGAGT	43
236	CCATGCGTCCTACAGCCTGCGACCTACGACCTGTATTCAGGAACTCAGGAATTATGAGT	59
237	GGAATGCTGGAAATTGGAATTATGAGT	27
238	CTCAGCATCTTGCTACTGGGCCGCAGGTCGTAGGTCGCAGGCTGTAGGGAATTATGAGT	59
239	AACGGCAGGAACTGAATCCCGAATTCGTCGCTGGCTGACAGC	42
240	AACGGCAGGAATTTTTTTTCCAGACATCCGACATTTTACTATTTGAGACCTGTGGCCAG	59
241	AACGGCAGGAAAGGTCTCACGTTTCATGGCTTTCAGCTGTCAGATGAGAAACAAGGGGT	59
242	AACGGCAGGAACTTAATTTCTGGCCACGGGGTACAAGGATATA	43
243	AACGGCAGGAACCTATGGCCTTTTGCATAGAACGACAGATGAT	43
244	AACGGCAGGAACACTGATGTCCAGCGGTGAAAGCCATGAAACG	43
245	AACGGCAGGAACTAGTGGCCTACTGATGAGATCTAAAGTTTAC	43
246	AACGGCAGGAATAGATCTCAGATCTTTGATCATATATCATCTGTAGGTGATGACTGAGA	59
247	AACGGCAGGAATGCGATCCGTAAACTTCGCGATTGGCGATGGC	43
248	AACGGCAGGAACATCAGGTCCCTGAGC	27
249	AACGGCAGGAAATTAGCATACAGCGTTATATGATCAAAGATCT	43

250	AACGGCAGGAACGTTTTACGACAAACTACAGTCCACTGGCCGTTGCGACGCGTGTAGCA	59
251	AACGGCAGGAACTCAAAGTCCGATTTATTATACGAATTAGAGA	43
252	CCAGATACCAAGTACCTGTGTTGAATAAACGAGCAAGGGTCAC	43
253	AACCAACTATCCCCTAGAAAATTAGGTACTTGGTATCTGGATGCCGCGGCAAGGGTCAC	59
254	GATTTGACCTAAACCCAAATGCGGTACGTGGTGCAAGGGTCAC	43
255	TAATTTTCTAGACACCGCATAGAGCACTGAGTGCAAGGGTCAC	43
256	GCTCGACGCTTAACGCAAATAGCGAAACGCGGGCAAGGGTCAC	43
257	CCGCATTTCGCGGCATACTATAGACAGTAGACGCAAGGGTCAC	43
258	CGTGTCCCCACGATTCAATACTGAGCGTTAAGCGTCGAGCGCCGGGCAGCAAGGGTCAC	59
259	CACCCCACTCATTCACTTGAAAAACGACGAGTGCAAGGGTCAC	43
260	TCAGTATTCTGTGCATGGTCTCGAAGAAAACAGCAAGGGTCAC	43
261	ACAAGTTTCGGCGCCCAACGTGCACCATTGCGGATAACAAGATACAGTGCAAGGGTCAC	59
262	TTTTTCAATGCCCGGCATACGGTAACAGGACTGCAAGGGTCAC	43
263	TAAACTAAGATCGGCGGCAAGGGTCAC	27
264	ACAATCGTCAATCGACAAACTTGTGACTAGAGGCAAGGGTCAC	43
265	TGGAATCCTCCGTCACGTTTAAGGGGACGTCGGACAACAGAAAAATAAGCAAGGGTCAC	59
266	AACGGCAGGAAGGTATCCTGGTACTCAGTAAAACGTAAATCGGACTTTGAGGCGGACAG	59
267	TCCTCATCTCCTTTCAGATGAAGAGTAAGGAG	32
268	AACCTACTATCTTCTAGAGAGCAAAGAGTTCG	32
269	AACTGTTTCGATTCATAGGCAGTGGCAAAGAT	32
270	TTTTTTTTCGAACTCTGGGGTGGATTTTTTTT	32
271	TTTTTTTTTATTTCCCGATGAGGATTTTTTTT	32
272	TTTTTTTTATCTTTGCAGTAGGTTTTTTTTTT	32
273	CTTCTTCCCCGCTTGCGGATTGCAGGGAAAAGCTCTAAGTCGTATGCC	48
274	ATCACACCAGTAACCAGGATCGCAGGGAGGCAGAGGGAGTTAAGAGGT	48
275	CCCCCCTCTTCTCACCTCCCTTACTCTCTAATTAACGGGTGAGAGGAT	48
276	CCCTCCCCGGCATACGACTTAGAGCTTTTCCCGGGGGGCAAAGGGGAG	48
277	TCAATCCTGTCCCGTCTAATCATTTACCTTTCGTAAGGGAGGTGAGAA	48
278	CCTACCTTCCTTCCCCATCCCTTTCTCCCCTTAGGAGGTTGGTAAAAG	48
279	ATTTCTCGCTCTTCGGAATGATTAGACGGGACAGGATTGAAGAACGGC	48
280	TCTCTCCATTTCCTTTACTGATTGGGGTTAAGGATTAGGACTTTGCAG	48
281	CCCCACCACTTTTACCCGAGAAATATGAGAGTTATGATTGGGTAAGCA	48
282	CAATCATAACTCTCATGGGGAGGGCTTATTTAGGAGCGGGTTGAAGGG	48
283	CTCTCTACACCTTTTCAAGGTGGGACGGAGAGGGTGAGTGTGAAAGGA	48
284	CACTGCCTTCCAACGCTCTCTTTCCCCTCACTTGGTGGGGGAATGTAG	48
285	TCCACCCCCCCTTCAACCCGCTCCTAAATAAGAAGGTAGGAAGTGAAG	48
286	TACCCTTCGCAACCCACTTGTCTCGCCGTTCTGAAAGAGAGCGTTGGA	48
287	TTTTTTTTCTCCTTACTCTTCATCCTTCACTTGGAGCAGATAGAAGAT	48
288	TTGCTCTCTGCTTACCGAGACAAGTGGGTTGCGAAGGGTATTTTTTTT	48
289	ATCACTCTACTTTCCAAGCAGTGCATGAATCGAAACAGTTTTTTTTTT	48

290	CTATCATTCTATAACCTTGGCTGCTAGCTGCGTGACGCACGAAAGGTA	48
291	TGCCCCCCGCCATCGCAGAAGCGATTTTTTTT	32
292	ACCCGTTAACCTTCTTAATGATAGGCAAGCGGGGAAGAAGCCGAAGAG	48
293	AACCTCCTATCCTCTCGGTGTGATAAAGGAAATGGAGAGAGAAAAGGT	48
294	CACTCACCCTCTCCGTCCCACCTTACCTCTTAAAAGGGATGGGGAAGG	48
295	GCACTGCTCTGCAAAGTCCTAATCCTTAACCCGAGGGGGGAGTGAGGG	48
296	TCTGCTCCCTACATTCGTAGAGAGTGGAAAGTAGAGTGATGGGAAATA	48
297	ACTCCCTCTGCCTCCC	16
298	ACTCCCTCTGCCTCCC	16
299	GGTATCCTGGTACTCAGTAAAACGTAAATCGGACTTTGAGGCGGACAG	48
300	GCATAGTCTTTTTTTTTTTTTTTTTCTCTGGGTATAAGTGTAGGCGATA	48
301	TACCTGAGTCTCAAGCTGCTGATATTTTTTTTTTTTTTTTGGGGGGAT	48
302	GTTGCCAGTCGTCTGCCGGCTACCTTTTTTTTTTTTTTTTGGGTTTTG	48
303	ATTACCGATTGCGACAGGGCTTTGGGCTTCGAATGTCTAAGCGCATGT	48
304	CGTAATCAAAAGTTGTCTACGGCCCCGACCTGCCTATAAAAAGAGGGG	48
305	TTTGTCTGTGTTCTGCGAGCCATATGTCACTAGAGTACGAGTTCATCG	48
306	CGGAAATCGTTACTAATGCGTGCTGCGCGGCGCGCAACGGTTTTTTTT	48
307	TCCCTAACCAAAACCCGACTATGCTGTCGCAA	32
308	ACTCCCTCTGCCTCCCCTAAAGCCGGAACTGG	32
309	CAATCAGTCTGTCCGCGGTAGCCGGCAGACGA	32
310	GCTACCGCTATCGCCTAGAGGCCGAATGCGTG	32
311	TCGTACTCGGTTCCAGTGGAGAGAGAAAAGGT	32
312	TTAGACATTCGAAGCCCTGGCAACTAGTGACA	32
313	GTCGAATCCGATGAACGTCTGGCGTTAGTAAC	32
314	CACTCACCCTCTCCGTCAAGATCAGGATCAAT	32
315	GCACTGCTCTGCAAAGTATGGCTCGCAGAACA	32
316	CCGGCGTGCAACCGCCAGAGTGATGGGAAATA	32
317	TACGCGACTTTTTTTT	16
318	GGCTTTAGTTTTTTTT	16
319	TTTTTTTTGGTGTCCG	16
320	GCACTAGCATCCCCCC	16
321	AACCTCCTATCCTCTCGGTGTGATAAAGGAAA	32
322	CCCACCTTACCTCTTAAAAGGGATGGGGAAGG	32
323	TCCTAATCCTTAACCCGAGGGGGGAGTGAGGG	32
324	TCTGCTCCCTACATTCGTAGAGAGTGGAAAGT	32
325	CGTGCTCACACATGCCGGCATCAGAGTTTGGG	32
326	GCCTCAGTTATCGACCGATTTCCGACTGTCAT	32
327	CCGTTGCGCGCCGCGCCAGACAAAAAGCATTA	32
328	TTTTTTTTATGACAGTTGAGCACGTTTTTTTT	32
329	TTTTTTTTTAATGCTTCACGCCGGTTTTTTTT	32
330	TTTTTTTTTCTGCGTTACTGAGGCTTTTTTTT	32
331	ACACTTATACCCAGAGGTCGCGTAACCGTGTCGGAGAAGGAGTGGCTA	48
332	CAAAGCCCCGGACACCTATCAGCAGCTTGAGACTCAGGTACAGGTCGG	48
333	TGATCTTGCACGCATTCGGCCTCTCCAGTTCCGTTAGGGACTGGAACC	48

334	TCTCTCACTAGCCACTCCTTCTCCGACACGGTGCTAGTGCTAAAAGGA	48
335	TTTGACGCCCCCTCTTTTTATAGGTCCTTTTATCGGTAATTTGAGCGC	48
336	CGCCAGACGCGCTCAAGCGGTAGCGGGGCTGAAGACTAAGGGCATGTG	48
337	CTTAGTCTTCAGCCCCGTGAGAGATGGTGGTAGGTGTATGAGGGTCAA	48
338	AGCACGCAACATGCGCGGCCGTAGACAACTTTTGATTACGAACGCAGA	48
339	TTTTTTTTCCCAAACTCTGATGCCATTGATCCGATTCGACGGCGGTTG	48
340	TTTTTTTTTTGACCCTCATACACCTACCACCAGCGTCAAAGGTCGATA	48
341	GGTATCCTGGTACTCAGTAAAACGTAAATCGGACTTTGAGTTTTTTTT	48
342	CATCAGGTCCCTGAGCTCTGAATCCCGAATTCGTCGCTGGCTGACAGC	48
343	CTGTATTTCAGTACTCGTTAACAGACGGCCAGTGGACTGTAGTTTGTC	48
344	CCAGATACCAAGTACCTGTGTTGAATAAACGATAAACTAAGATCGGCG	48
345	TTTTTTTTCCAGACATCCGACATTTTACTATTTGAGACCTGTGGCCAG	48
346	ACAAGTTTCGGCGCCCAACGTGCACCATTGCGGATAACAAGATACAGT	48
347	AACCAACTATCCCCTAGAAAATTAGGTACTTGGTATCTGGATGCCGCG	48
348	AGGTCTCACGTTTCATGGCTTTCAGCTGTCAGATGAGAAACAAGGGGT	48
349	CGTGTCCCCACGATTCAATACTGAGCGTTAAGCGTCGAGCGCCGGGCA	48
350	TAGATCTCAGATCTTTGATCATATATCATCTGTAGGTGATGACTGAGA	48
351	ACGCCGCACAACATATGCGCGTGGCGCAATGGTGCACGTTGGGCGCCG	48
352	CGTTTTACGACAAACTACAGTCCACTGGCCGTTGCGACGCGTGTAGCA	48
353	TAATTTTCTAGACACCGCATAGAGCACTGAGT	32
354	CCTATGGCCTTTTGCATAGAACGACAGATGAT	32
355	TCAGTATTCTGTGCATGGTCTCGAAGAAAACA	32
356	CACTGATGTCCAGCGGTGAAAGCCATGAAACG	32
357	GCTCGACGCTTAACGCAAATAGCGAAACGCGG	32
358	ATTAGCATACAGCGTTATATGATCAAAGATCT	32
359	CCGCATTTCGCGGCATACTATAGACAGTAGAC	32
360	CTAGTGGCCTACTGATGAGATCTAAAGTTTAC	32
361	TTTTTCAATGCCCGGCATACGGTAACAGGACT	32
362	GATTTGACCTAAACCCAAATGCGGTACGTGGT	32
363	CTTAATTTCTGGCCACGGGGTACAAGGATATA	32
364	CACCCCACTCATTCACTTGAAAAACGACGAGT	32
365	TGCGATCCGTAAACTTCGCGATTGGCGATGGC	32
366	ACAATCGTCAATCGACAAACTTGTGACTAGAG	32
367	CTCAAAGTCCGATTTATTATACGAATTAGAGA	32
368	CCCGCCCCTTTTTTTTTTTTTTTTGGGGACCGCGAGATATAAGACGTA	48
369	GCGGCCCACAATTTCCAGCATTCCTTTTTTTTTTTTTTTTGGAACTTA	48
370	CCTACCGACTCATAATTGGCAATAAGTTAAAAGCGGCGGGGTCTAGGA	48
371	CCATGCGTCCTACAGCCTGCGACCTACGACCTGTATTCAGGAACTCAG	48
372	TGTCACATCACGTCTCTGCCAGGGATTATGAGTCGGTAGGCGGTGATT	48
373	GTCGGCACCAACATCCGGATAACGGTGACTGGTGGCTGGTCGTGATTT	48
374	CTCAGCATCTTGCTACTGGGCCGCAGGTCGTAGGTCGCAGGCTGTAGG	48
375	GGGGCGGGAATCTAGG	16
376	GGGCTGTGGAGCATTG	16
377	GGAATGCTGGAAATTG	16

378	CACAGCCCTTTTTTTTTTTTTTTTGGGCGGAT	32
379	ACCAGCCACCAGTCACTCAGGGGTCCAGAAGA	32
380	CCCTGGCACCTAGATTTACGGGTGACAGGTAA	32
381	CGTTATCCCAATGCTCGGGCTGCGGGTCATAG	32
382	CATAATACTAAGAGCTATGGGTTTTTTTTGGCTAGTAGATTGTTTGGAGTCAG	53
383	GCCCTTCCTAAGAGCTATGGGTTTTTTTTGGAGACCAAAGAATGTTGAGCAGG	53
384	ATTTAGCCCGGAGCCCTGTGCTCCCAACAGAGGCAGATTTTTTTTGGTATTGT	53
385	TTAGAACTCCGATACCCGATATCCCAACAGAGGCAGATTTTTTTTGGGTCCGG	53
386	TACTGCCCTAAGAGCTATGGG	21
387	CGGAACCCTAAGAGCTATGGG	21
388	GGCGGCTAAAGTGTACTCCTAAGAGCTATGGG	32
389	GGCGGCTAAAGAGGCCCCCCAACAGAGGCAGATTTTTTTTGGGCCGAT	48
390	TCGATCTCCAACAGAGGCAGATTTTTTTTGGGACAAA	37
391	ATTTGCGCCAACAGAGGCAGATTTTTTTTGAGGACAT	37
392	TTTTTTTTTATATCCTATAAATTACCCATAGCTCTTA	37
393	TTTTTTTTTCTCTAATTAACGGGTTCTGCCTCTGTTG	37
394	TTTTTTTTACCCGGCTGTTTGCGGCCCATAGCTCTTA	37
395	TTTTTTTTTAATCTATGGCTTGAATCTGCCTCTGTTG	37
396	TTTTTTTTAACAGCTTATCCAGGACCCATAGCTCTTA	37
397	TTTTTTTTTTACGTCCGTCAAATCCCCATAGCTCTTA	37
398	TTTTTTTTACCACGTAACCGATGTCCCATAGCTCTTA	37
399	TTTTTTTTCTCTAGTCCAAGGTACTCTGCCTCTGTTG	37
400	ACACCCTTGGCTTCCCTGGCGTATGTCGATTGACGATTGTTCTGCCTCTGTTG	53
401	CTATCATTCTATAACCTTGGCTGCTAGCTGCGTGACGCACTCTGCCTCTGTTG	53
402	CCCGCCCCTTTTTTTTTTTTTTTTGGGGACCGCGATGTGTCGC	43
403	CCTACCGACTCATAATTGGCAATAAGTTAAAAGCGGCGGGGTCTAGGACGATGTGTCGC	59
404	GGGGCGGGAATCTAGGCGATGTGTCGC	27
405	TGTCACATCACGTCTCTGCCAGGGATTATGAGTCGGTAGGCGGTGATTCGATGTGTCGC	59
406	CACAGCCCTTTTTTTTTTTTTTTTGGGCGGATCGATGTGTCGC	43
407	CCCTGGCACCTAGATTTACGGGTGACAGGTAACGATGTGTCGC	43
408	ACCAGCCACCAGTCACTCAGGGGTCCAGAAGACGATGTGTCGC	43
409	GGGCTGTGGAGCATTGCGATGTGTCGC	27
410	GTCGGCACCAACATCCGGATAACGGTGACTGGTGGCTGGTCGTGATTTCGATGTGTCGC	59
411	GCGGCCCACAATTTCCAGCATTCCTTTTTTTTTTTTTTTTGGAACTTACGATGTGTCGC	59
412	CGTTATCCCAATGCTCGGGCTGCGGGTCATAGCGATGTGTCGC	43
413	CCATGCGTCCTACAGCCTGCGACCTACGACCTGTATTCAGGAACTCAGCGATGTGTCGC	59
414	GGAATGCTGGAAATTGCGATGTGTCGC	27
415	CTCAGCATCTTGCTACTGGGCCGCAGGTCGTAGGTCGCAGGCTGTAGGCGATGTGTCGC	59
416	CCCGCCCCTTTTTTTTTTTTTTTTGGGGACCGCGAGATATAAGACGTA	48
417	CTCCGGCCTTCTGATCCCCGCTGCCTGCCACCGAGCGCTGAGAACCGT	48
418	TAAAACCTCGGGCCCCGGGCAGCTGGCGGCGAACGGGAGTCCGGCGAT	48
419	GCGGCCCACAATTTCCAGCATTCCTTTTTTTTTTTTTTTTGGAACTTA	48
420	CCTACCGACTCATAATTGGCAATAAGTTAAAAGCGGCGGGGTCTAGGA	48
421	TTTTTTTTCACCCAGTGCCCTATGCCCTTGATGGGGCGCTGCAGGATG	48

422	TTTTTTTTATACCATACAACTCATGTTTCCTACGCAGGGAGAGTAGAA	48
423	TTTTTTTTCCGCGGCTCCCAGCCCACTGACCCAGCTAAGCCAGGTCAG	48
424	CCATGCGTCCTACAGCCTGCGACCTACGACCTGTATTCAGGAACTCAG	48
425	TGTCACATCACGTCTCTGCCAGGGATTATGAGTCGGTAGGCGGTGATT	48
426	AGCGCCCCGTACTACATACATCCTACGGTTCTGACGGTACTATACAGG	48
427	GTCGGCACCAACATCCGGATAACGGTGACTGGTGGCTGGTCGTGATTT	48
428	CAATTGCCGCTAGCCCCCGAGTCCCGCACGCTGCGTGGATTGGACGGG	48
429	CTCAGCATCTTGCTACTGGGCCGCAGGTCGTAGGTCGCAGGCTGTAGG	48
430	GCTTGCAAATCGCCGGACTCCCGTTCGCCGCCAGTTCTAAGGACTTAA	48
431	TGTACTCCTTTTTTTT	16
432	GGGGCGGGAATCTAGG	16
433	CAGCGCTCCAAGTCCC	16
434	GGGCTGTGGAGCATTG	16
435	GCTTGTCCATCGGCCC	16
436	GGAATGCTGGAAATTG	16
437	AGCTGCCCAGTATCCC	16
438	ATTCACCCTTTTTTTTTTTTTTTTGGGACTTG	32
439	CACAGCCCTTTTTTTTTTTTTTTTGGGCGGAT	32
440	AGGCCCCCTTTTTTTTTTTTTTTTGGGCCGAT	32
441	CCCTGGCACCTAGATTTACGGGTGACAGGTAA	32
442	CCTTGGGTGCCCCGCAGGACAAGCAGCGTGCG	32
443	CGTTATCCCAATGCTCGGGCTGCGGGTCATAG	32
444	CTCGGCCATCCGCTGTAGGATGTATGTAGTAC	32
445	ACCAGCCACCAGTCACTCAGGGGTCCAGAAGA	32
446	CCATTCCACGGACCTCGGACTCGGGGGCTAGC	32
447	GTCTTGGCCTGACCTGGATGTTAAGTGACGGA	32
448	GTACCTCGCATCCTGCATGGTTGAGTGAACGA	32
449	TGTGTCCACATTCAATGTGAGATGCAGGTTGC	32
450	ATCCGCGCTACGTATCGGCAGGAGCCAACGGT	32
451	TTCGCGTGCCACACGTACGCATGGCGTCGGGT	32
452	CTTCCGATCGCCCGTAAATAGTGGTACGAAGT	32
453	ATCCGCAACTGCCATCGACGAATGACGTGTGGCACGCGAATTTTTTTT	48
454	CCCATGTTCTTGCCCTAAGTCAAGAGACACAAGGCGTTTGTTTTTTTT	48
455	CCCTAGTTCCCAGATCTTGCAAGCTACGGGCGATCGGAAGTTTTTTTT	48
DUT1-1	TTTTTTTTTTTCGCAGGTGGAGTTTTTTTTTTGGTTGTTGGATTTCA	47
DUT1-2	TTTTTTTTTGTCTCCGTGGAAGTATTTTTTTTGGTTGTTGGATTTCA	47
DUT1-3	TTTTTTTTAACCCGAGGCGAGTGCTTTTTTTTGGTTGTTGGATTTCA	47
DUT1-4	TTTTTTTTGACATCGCGCCAAGACTTTTTTTTGGTTGTTGGATTTCA	47
DUT1-5	TTTTTTTTACCGTTGGGATAAATGTTTTTTTTGGTTGTTGGATTTCA	47
DUT1-6	TTTTTTTTGCTCTTGTTGGACACATTTTTTTTGGTTGTTGGATTTCA	47
DUT1-7	TTTTTTTTTCGTTCACGAGACATATTTTTTTTGGTTGTTGGATTTCA	47
DUT1-8	TTTTTTTTCGTTGCCTGAACTTGGTTTTTTTTGGTTGTTGGATTTCA	47
DUT1-9	TTTTTTTTTCCGTCACGTTTAAGGTTTTTTTGGTTGTTGGATTTCA	47
DUT1-10	TTTTTTTTCACGCTACCGAGGTACTTTTTTTTGGTTGTTGGATTTCA	47

DUT1-11	TTTTTTTGCAACCTGTGGGCGTGTTTTTTTGGTTGTTGGATTTCA	47
DUT1-12	TTTTTTTTTACCGGATGCGCGGATTTTTTTTTGGTTGTTGGATTTCA	47
DUT1-13	TTTTTTTTACTTCGTAACATATTGTTTTTTTGGTTGTTGGATTTCA	47
DUT1-14	TTTTTTTTTCAATACAGTCTGAACTTTTTTTTGGTTGTTGGATTTCA	47
DUT1-15	TTTTTTTTACCCGACGTGTGGTTATTTTTTTTGGTTGTTGGATTTCA	47
DUT2-1	TTTTTTTTTTTCGCAGGTGGAGTTTTTTTTTGGCTGGCAGGATGCT	47
DUT2-2	TTTTTTTTGTCTCCGTGGAAGTATTTTTTTTGGCTGGCAGGATGCT	47
DUT2-3	TTTTTTTTAACCCGAGGCGAGTGCTTTTTTTTGGCTGGCAGGATGCT	47
DUT2-4	TTTTTTTTGACATCGCGCCAAGACTTTTTTTTGGCTGGCAGGATGCT	47
DUT2-5	TTTTTTTTACCGTTGGGATAAATGTTTTTTTGGCTGGCAGGATGCT	47
DUT2-6	TTTTTTTTGCTCTTGTTGGACACATTTTTTTTGGCTGGCAGGATGCT	47
DUT2-7	TTTTTTTTTCGTTCACGAGACATATTTTTTTTGGCTGGCAGGATGCT	47
DUT2-8	TTTTTTTTCGTTGCCTGAACTTGGTTTTTTTTGGCTGGCAGGATGCT	47
DUT2-9	TTTTTTTTTCCGTCACGTTTAAGGTTTTTTTGGCTGGCAGGATGCT	47
DUT2-10	TTTTTTTTCACGCTACCGAGGTACTTTTTTTTGGCTGGCAGGATGCT	47
DUT2-11	TTTTTTTTGCAACCTGTGGGCGTGTTTTTTTTGGCTGGCAGGATGCT	47
DUT2-12	TTTTTTTTTACCGGATGCGCGGATTTTTTTTTGGCTGGCAGGATGCT	47
DUT2-13	TTTTTTTTACTTCGTAACATATTGTTTTTTTGGCTGGCAGGATGCT	47
DUT2-14	TTTTTTTTTCAATACAGTCTGAACTTTTTTTTGGCTGGCAGGATGCT	47
DUT2-15	TTTTTTTTACCCGACGTGTGGTTATTTTTTTTGGCTGGCAGGATGCT	47
DUB1-1	GTCGGTTCACGGAGCTTTTTTTTTTTCGCAGGTGGAGTTTTTTTTT	47
DUB1-2	GTCGGTTCACGGAGCTTTTTTTTGTCTCCGTGGAAGTATTTTTTTT	47
DUB1-3	GTCGGTTCACGGAGCTTTTTTTTAACCCGAGGCGAGTGCTTTTTTTT	47
DUB1-4	GTCGGTTCACGGAGCTTTTTTTTGACATCGCGCCAAGACTTTTTTTT	47
DUB1-5	GTCGGTTCACGGAGCTTTTTTTTTACCGTTGGGATAAATGTTTTTTTT	47
DUB1-6	GTCGGTTCACGGAGCTTTTTTTTGCTCTTGTTGGACACATTTTTTTT	47
DUB1-7	GTCGGTTCACGGAGCTTTTTTTTTCGTTCACGAGACATATTTTTTTT	47
DUB1-8	GTCGGTTCACGGAGCTTTTTTTTCGTTGCCTGAACTTGGTTTTTTTT	47
DUB1-9	GTCGGTTCACGGAGCTTTTTTTTTCCGTCACGTTTAAGGTTTTTTTT	47
DUB1-10	GTCGGTTCACGGAGCTTTTTTTTCACGCTACCGAGGTACTTTTTTTT	47
DUB1-11	GTCGGTTCACGGAGCTTTTTTTTGCAACCTGTGGGCGTGTTTTTTTT	47
DUB1-12	GTCGGTTCACGGAGCTTTTTTTTTACCGGATGCGCGGATTTTTTTTT	47
DUB1-13	GTCGGTTCACGGAGCTTTTTTTTACTTCGTAACATATTGTTTTTTTT	47
DUB1-14	GTCGGTTCACGGAGCTTTTTTTTTCAATACAGTCTGAACTTTTTTTT	47
DUB1-15	GTCGGTTCACGGAGCTTTTTTTTACCCGACGTGTGGTTATTTTTTTT	47
DUB2-1	GACCGAGTTACTGTTTTTTTTTTTTTCGCAGGTGGAGTTTTTTTTT	47
DUB2-2	GACCGAGTTACTGTTTTTTTTTTTGTCTCCGTGGAAGTATTTTTTTT	47
DUB2-3	GACCGAGTTACTGTTTTTTTTTTAACCCGAGGCGAGTGCTTTTTTTT	47
DUB2-4	GACCGAGTTACTGTTTTTTTTTTGACATCGCGCCAAGACTTTTTTTT	47
DUB2-5	GACCGAGTTACTGTTTTTTTTTTACCGTTGGGATAAATGTTTTTTTT	47
DUB2-6	GACCGAGTTACTGTTTTTTTTTTGCTCTTGTTGGACACATTTTTTTT	47
DUB2-7	GACCGAGTTACTGTTTTTTTTTTTCGTTCACGAGACATATTTTTTTT	47
DUB2-8	GACCGAGTTACTGTTTTTTTTTTCGTTGCCTGAACTTGGTTTTTTTT	47
DUB2-9	GACCGAGTTACTGTTTTTTTTTTTCCGTCACGTTTAAGGTTTTTTTT	47

DUB2-10	GACCGAGTTACTGTTTTTTTTTTCACGCTACCGAGGTACTTTTTTTT	47
DUB2-11	GACCGAGTTACTGTTTTTTTTTTGCAACCTGTGGGCGTGTTTTTTTT	47
DUB2-12	GACCGAGTTACTGTTTTTTTTTTTACCGGATGCGCGGATTTTTTTTT	47
DUB2-13	GACCGAGTTACTGTTTTTTTTTTACTTCGTAACATATTGTTTTTTTT	47
DUB2-14	GACCGAGTTACTGTTTTTTTTTTTCAATACAGTCTGAACTTTTTTTT	47
DUB2-15	GACCGAGTTACTGTTTTTTTTTTACCCGACGTGTGGTTATTTTTTTT	47
DUB3-1	GCAGCTCGTGGACCATTTTTTTTTTTCGCAGGTGGAGTTTTTTTTTT	47
DUB3-2	GCAGCTCGTGGACCATTTTTTTTTGTCTCCGTGGAAGTATTTTTTTT	47
DUB3-3	GCAGCTCGTGGACCATTTTTTTTAACCCGAGGCGAGTGCTTTTTTTT	47
DUB3-4	GCAGCTCGTGGACCATTTTTTTTGACATCGCGCCAAGACTTTTTTTT	47
DUB3-5	GCAGCTCGTGGACCATTTTTTTTACCGTTGGGATAAATGTTTTTTTT	47
DUB3-6	GCAGCTCGTGGACCATTTTTTTTGCTCTTGTTGGACACATTTTTTTT	47
DUB3-7	GCAGCTCGTGGACCATTTTTTTTTCGTTCACGAGACATATTTTTTTT	47
DUB3-8	GCAGCTCGTGGACCATTTTTTTTCGTTGCCTGAACTTGGTTTTTTTT	47
DUB3-9	GCAGCTCGTGGACCATTTTTTTTTCCGTCACGTTTAAGGTTTTTTTT	47
DUB3-10	GCAGCTCGTGGACCATTTTTTTTCACGCTACCGAGGTACTTTTTTTT	47
DUB3-11	GCAGCTCGTGGACCATTTTTTTTGCAACCTGTGGGCGTGTTTTTTTT	47
DUB3-12	GCAGCTCGTGGACCATTTTTTTTTACCGGATGCGCGGATTTTTTTTT	47
DUB3-13	GCAGCTCGTGGACCATTTTTTTTACTTCGTAACATATTGTTTTTTTT	47
DUB3-14	GCAGCTCGTGGACCATTTTTTTTTCAATACAGTCTGAACTTTTTTTT	47
DUB3-15	GCAGCTCGTGGACCATTTTTTTTACCCGACGTGTGGTTATTTTTTTT	47

15. References

S1 N. C. Seeman, *J. Biomol. Struct. Dyn.*, 1990, **8**, 573-581.

S2 B.Wei, M. Dai and P.Yin, *Nature*, 2012, **485**, 623-627.

S3 Y. Ke, L. L. Ong, W. M. Shih and P. Yin, *Science*, 2012, **338**, 1177-1183.
